# Quaternary Ammonium Palmitoyl Glycol Chitosan (GCPQ) Loaded with Platinum-Based Anticancer Agents—A Novel Polymer Formulation for Anticancer Therapy

**DOI:** 10.3390/ph16071027

**Published:** 2023-07-19

**Authors:** Yvonne Lerchbammer-Kreith, Michaela Hejl, Nadine S. Sommerfeld, Xian Weng-Jiang, Uchechukwu Odunze, Ryan D. Mellor, David G. Workman, Michael A. Jakupec, Andreas G. Schätzlein, Ijeoma F. Uchegbu, Mathea S. Galanski, Bernhard K. Keppler

**Affiliations:** 1 Institute of Inorganic Chemistry, Faculty of Chemistry, University of Vienna, Waehringer Strasse 42, 1090 Vienna, Austria; 2 School of Pharmacy, University College London, Brunswick Square 29-39, London WC1N 1AX, UK; 3 Research Cluster “Translational Cancer Therapy Research”, University of Vienna, Waehringer Strasse 42, 1090 Vienna, Austria

**Keywords:** platinum(IV) complexes, quaternary ammonium glycol chitosan (GCPQ), anticancer, drug delivery

## Abstract

Quaternary ammonium palmitoyl glycol chitosan (GCPQ) has already shown beneficial drug delivery properties and has been studied as a carrier for anticancer agents. Consequently, we synthesised cytotoxic platinum(IV) conjugates of cisplatin, carboplatin and oxaliplatin by coupling via amide bonds to five GCPQ polymers differing in their degree of palmitoylation and quaternisation. The conjugates were characterised by ^1^H and ^195^Pt NMR spectroscopy as well as inductively coupled plasma mass spectrometry (ICP-MS), the latter to determine the amount of platinum(IV) units per GCPQ polymer. Cytotoxicity was evaluated by the MTT assay in three human cancer cell lines (A549, non-small-cell lung carcinoma; CH1/PA-1, ovarian teratocarcinoma; SW480, colon adenocarcinoma). All conjugates displayed a high increase in their cytotoxic activity by factors of up to 286 times compared to their corresponding platinum(IV) complexes and mostly outperformed the respective platinum(II) counterparts by factors of up to 20 times, also taking into account the respective loading of platinum(IV) units per GCPQ polymer. Finally, a biodistribution experiment was performed with an oxaliplatin-based GCPQ conjugate in non-tumour-bearing BALB/c mice revealing an increased accumulation in lung tissue. These findings open promising opportunities for further tumouricidal activity studies especially focusing on lung tissue.

## 1. Introduction

Platinum(II) complexes such as the worldwide approved drugs cisplatin, carboplatin and oxaliplatin are first-line anticancer agents against different tumour types and these compounds still play an essential role in modern cancer chemotherapy [1,2,3]. However, their clinical success is limited due to severe adverse effects such as nephrotoxicity and neurotoxicity as well as intrinsic and acquired therapy resistance [4,5]. The introduction of additional ligands in the axial position leads to platinum(IV) complexes with higher kinetic inertness which enables the promising possibility of overcoming platinum(II)-based drawbacks [6,7]. Platinum(IV) prodrugs demonstrate their cytotoxic activity after the reduction to the corresponding platinum(II) counterparts by releasing their axial ligands [8,9]. Supportive conditions for the required reduction are provided by the acidic and oxygen-deficient milieu of tumour tissue, resulting in a potentially improved selectivity for platinum(IV) complexes towards cancerous tissue [10,11]. Additionally, the octahedral structure of platinum(IV) agents can be exploited for introducing targeting moieties as well as enabling the fine-tuning of pharmacokinetic properties [12,13].

The attachment of platinum(IV) compounds to different types of nanoparticles via axial ligands is a promising approach for drug delivery purposes [14,15,16]. In particular, passive tumour targeting by exploiting the enhanced permeability and retention effect (EPR effect) is attracting more and more interest. The EPR effect is a characteristic of tumour tissue based on the existence of large gaps between the endothelial cells in combination with dysfunctional lymphatic drainage. Thereby, the penetration and accumulation in tumour tissue by molecules in the nanometre range is facilitated [17,18].

Promising nanoparticles with excellent drug delivery properties can be found in the class of chitosan, a naturally obtained polysaccharide [19,20,21]. Chitosan is only soluble at acid pH in aqueous media; however, modifications lead to derivatives such as *N*-palmitoyl-*N*-monomethyl-*N*,*N*-dimethyl-*N*,*N*,*N*-trimethyl-6-*O*-glycolchitosan (GCPQ) which self-assembles in aqueous media into stable colloids [22,23]. As an amphiphile, GCPQ spontaneously forms these micellar nano-sized clusters in aqueous media and is also able to facilitate the aqueous incorporation of lipophilic compounds by effectively solubilising them [24,25,26]. GCPQ functions as an improved transporter for hydrophobic and amphiphilic agents through the epithelium of the gastrointestinal tract and the cornea as well as to the brain via the nose-to-brain route. Due to the encapsulation inside GCPQ nanoparticles, drug degradation in the gastrointestinal tract can theoretically be prevented [25,27].

GCPQ is a non-toxic carrier that forms particles in the nanometre range; therefore, it is hypothesised that tumour targeting through passive diffusion caused by the EPR effect is possible. Consequently, the use of GCPQ polymers could build the basis for a promising drug delivery strategy for anticancer agents [28]. Recent in vitro experiments with GCPQ micelles serving as nanocarriers for selumetinib (AZD6244), an organic kinase inhibitor and anticancer agent, showed very promising results. In comparison to the free drug, GCPQ-based nanoparticles were superior in monolayer cell culture experiments and tumouroids in spite of their poor diffusion through the tumouroid tissue [29]. Furthermore, the lipophilic anticancer agent etoposide was encapsulated in GCPQ micelles, leading to enhanced cellular uptake in mammary cancer cells. Additionally, biodistribution studies in A431 xenografted mice showed a successful delivery of loaded GCPQ polymers to the solid tumour [30]. Additionally, GCPQ polymers loaded with the anticancer drug doxorubicin showed improved cellular uptake and increased cytotoxic activity compared to free doxorubicin. Furthermore, in vivo studies in a skin tumour model revealed enhanced accumulation in cancerous tissue, detected by fluorescence imaging [31].

Inspired by the promising use of GCPQ micelles as drug delivery systems, we developed and investigated GCPQ polymers loaded with cytotoxic platinum(IV) complexes. In analogy to a previously published study [19], platinum(IV) analogues of cisplatin, carboplatin and oxaliplatin were conjugated via amide bonds to GCPQ nanoparticles, differing in levels of palmitoylation and quaternisation. Characterisation of the conjugates was performed by NMR spectroscopy and inductively coupled plasma mass spectrometry (ICP-MS). IC_50_ values in three different human cancer cell lines were determined via the MTT assay. Finally, the biodistribution of an oxaliplatin-based GCPQ conjugate compared to its carrier-free platinum(IV) counterpart was investigated in non-tumour-bearing mice.

## 2. Results and Discussion

### 2.1. Synthesis

**Scheme 1:**
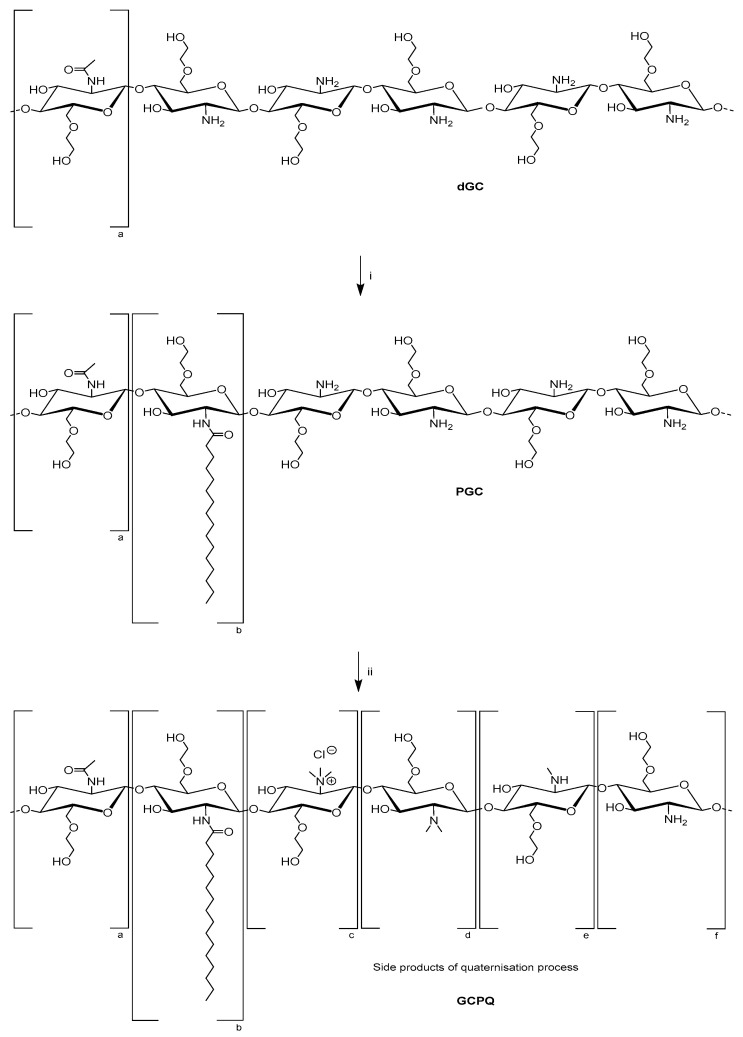
Synthetic steps for preparation of GCPQ polymers starting from degraded glycol chitosan (dGC), containing approximately 5% of acetylated monomers. (**i**) Palmitic acid *N*-hydroxysuccinimide (PNS), DMSO/triethylamine (TEA), (**ii**) sodium hydroxide, sodium iodide, methyl iodide, *N*-methyl-2-pyrrolidon (NMP), nitrogen atmosphere. Following monomers are shown: (a) acetylated GC, (b) palmitoylated GC, (c) quaternised GC, (d) dimethylamine GC, (e) methylamine GC, (f) GC.

The synthesis of GCPQ polymers was conducted according to procedures previously reported (Scheme 1) [24]. Starting from acidic degradation of glycol chitosan (MW ≈ 120 kDa) with hydrochloric acid, the obtained degraded glycol chitosan (dGC) was derivatised by reaction with palmitic acid *N*-hydroxysuccinimide (PNS). The formed palmitoyl glycol chitosan (PGC) was further treated with sodium hydroxide, sodium iodide and methyl iodide under a nitrogen atmosphere in order to produce quaternary ammonium groups. Besides quaternisation, di- and methylamine side chains were additionally formed, whereas some amino groups did not react at all. The corresponding levels of palmitoylation and quaternisation can be controlled by the amount of PNS and methyl iodide as well as reaction time. Consequently, five GCPQ polymers were synthesised, differing by the proportion of palmitoylated and quaternised monomers. These GCPQ polymers were synthesised in order to determine the effects of different palmitoylation and quaternisation levels on cytotoxicity and biodistribution. Polymers with a palmitoylation and quaternisation level of 7% will be described as **GCP7Q7**, whereas in **GCP21Q27**, the level of palmitoylation and quaternisation is 21% and 27%, respectively (Table 1).

Synthesis of platinum(IV) complexes **1**–**3** as well as conjugation to GCPQ polymer followed a synthetic pathway published previously [19].

**Scheme 2:**
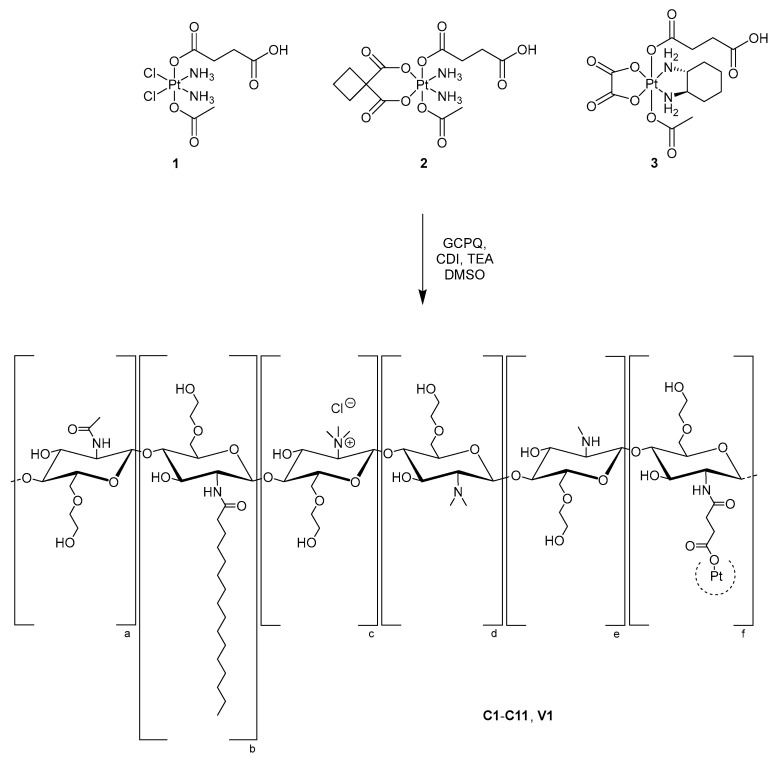
Illustration of the coupling reaction of platinum(IV) complexes **1**–**3** to five different GCPQ polymers resulting in conjugates **C1**–**C11**, **V1**.

Conjugation of platinum(IV) complexes to GCPQ was performed via amide bond formation between the acid group of platinum(IV) compounds and primary amines of GCPQ polymers, using 1,1′-carbonyldiimidazole (CDI) as a coupling agent (Scheme 2). The resulting conjugates **C1**–**C11**, **V1** (Table 2) were dialysed against Milli-Q water, treated with HCl to adjust a pH of approximately 3 and finally obtained by freeze-drying. The complex-to-polymer ratios affected the aqueous solubility of the conjugates; therefore, it was important to avoid an overload of GCPQ polymers with platinum(IV) complexes in order to prevent precipitation of the conjugates.

In particular, conjugates containing the cisplatin-based complex **1** as well as the carboplatin-based complex **2** tended to precipitate during the dialysis process. Thus, equivalents of GCPQ polymers were increased to prevent precipitation and significantly improved the solubility of **C1**–**C6**. Despite increased polymer equivalents, conjugation of complex **2** to **GCP7Q7** could not be successfully performed due to low water solubility and accompanying precipitation during dialysis.

### 2.2. Analysis

The different levels of palmitoylation and quaternisation of the five synthesised GCPQ polymers were determined via ^1^H NMR spectroscopy by comparing the ratios of terminal methyl protons of the palmitoyl moiety, the protons of the quaternary ammonium group and protons of the sugar backbone, respectively (Supporting Information, Figure S1) [24]. The synthesised GCPQ polymers featured 7–22 mol % palmitoylation and 7–33 mol % quaternisation, respectively. The molecular weights (MW) of the GCPQ polymers were measured by gel permeation chromatography with multiangle laser light scattering (GPC-MALLS) and they ranged from 9.6 to 18.0 kDa (Table 1).

^1^H and ^195^Pt NMR spectra were utilised for characterisation of conjugates **C1**–**C11**, **V1** confirming the successful attachment of platinum(IV) complexes to the GCPQ polymers. Prominent signals of the acetato ligand of the platinum(IV) complexes were detected in the ^1^H NMR spectra between 2.03 and 2.12 ppm, as well as multiplets of the succinato ligand between 2.46 and 2.72 ppm. Additionally, peaks, referring to the cyclobutane-1,1-dicarboxylato ligand of carboplatin-based complex **2** as well as the cyclohexane-1,2-diamine ligand of oxaliplatin analogue **3**, were found in the upfield region of the NMR spectra. Characteristic ^195^Pt signals between 2705 and 3509 ppm further proved the presence of the corresponding platinum(IV) species (Supporting Information, Figures S2–S8). The average platinum(IV) units per GCPQ polymer were calculated based on inductively coupled plasma mass spectrometry (ICP-MS) measurements and were further used for the calculation of molecular weights of the conjugates (Table 2).

Conjugates **C1**–**C11** displayed a different pattern of solubility, depending on the respective GCPQ polymer and platinum(IV) complex. In general, the following trend in the GCPQ influence could be observed, starting with the highest solubility for conjugates containing: **GCP22Q33** > **GCP21Q27** > **GCP21Q12** > **GCP7Q7**. This observation mostly corresponded to the ratio of the degree of quaternisation to palmitoylation (QPR, Table 1), a parameter for hydrophilicity of GCPQ polymers [25]. Furthermore, conjugates loaded with the oxaliplatin analogue **3** displayed the highest solubility, whereas conjugates featuring cisplatin (**1**) and carboplatin moieties (**2**) were less soluble (Supporting Information, Table S1).

### 2.3. Cytotoxicity

A comparison of cytotoxic potencies in three human cancer cell lines according to results from the MTT assay after 96 h exposure (Table 3 and Table 4, Supporting Information, Figures S9–S12) revealed certain patterns of cytotoxicity: While it has been shown previously that platinum(IV) derivatives of the established platinum(II) drugs show (by 1–2 orders of magnitude) diminished cytotoxicity due to their higher inertness as expected [19], coupling to GCPQ polymers usually compensates for most of this loss, and in the majority of cases even overcompensates it. This effect was most conspicuous for conjugates **C10** and **C11**. Taking into account the loading of approximately 7.3 and 11.0 platinum(IV) units per polymer, respectively, these two products were 96–286 times (Table 4, based on IC_50_ values, depending on the cell line) more potent than platinum(IV) derivative **3**. Additionally, they also outperformed their parent drug oxaliplatin, especially in CH1/PA-1 teratocarcinoma cells (by factors of 6.8 and 9.1) and the colon carcinoma cell line SW480 (by factors of 3.5 and 3.8), the latter representing a malignancy typically treated with this drug.

Relative to the palpably least potent of the parent drugs, i.e., carboplatin, conjugates **C7** and **C6** performed comparably well (factors of 2.5–8.8, depending on the cell line) or even better (factors of 10.5–20.3), respectively. In two of the three cell lines, however, the performance of these two conjugates may be explained by the cytotoxicity that the unloaded polymers exert by themselves, as reflected in the rather similar IC_50_ values of **C6** and **GCPQ21Q27** as well as **C7** and **GCP22Q33** in A549 and SW480 cells. Generally, substantial but unselective cytotoxic potencies were observed for the three unloaded polymers that were suitable for testing, with IC_50_ values mostly in the low micromolar range and a minor dependency on the cell line (i.e., no hypersensitivity of CH1/PA-1 cells, in contrast to all the platinum-containing substances except for oxaliplatin).

Nevertheless, most of the other comparisons revealed distinct increases in cytotoxicity as a result of platinum loading. Moreover, the factors by which potencies of conjugates **C8**, **C10** and **C11** exceeded those of the corresponding unloaded polymers (A549: >6.3, 22, 45; CH1/PA-1: 60, 444, 528; SW480: 24, 100, 119 times, respectively) suggest a fairly good correlation with the degree of platinum loading (1.7, 7.3 and 11.0 platinum(IV) units per polymer, respectively). Assessment of correlation remained incomplete, however, because precipitation of **GCP21Q12** in the test medium made a comparison with **C9** impossible.

Based on the promising results, we decided to further investigate an oxaliplatin-based conjugate in vivo due to its favourable combination of solubility and cytotoxicity. Despite accurate controlling of the reaction parameters, the exact resynthesis of GCPQ polymers and the following platinum(IV) loading is challenging. Therefore, conjugate **V1**, a close analogue of **C8**, was synthesised de novo with 1.5 platinum(IV) units per **GCP8Q10** polymer. The cytotoxic activity was tested in the murine mammary carcinoma cell line 4T1, revealing an IC_50_ value in the high nanomolar range (Table 5).

### 2.4. Biodistribution

**Figure 1:**
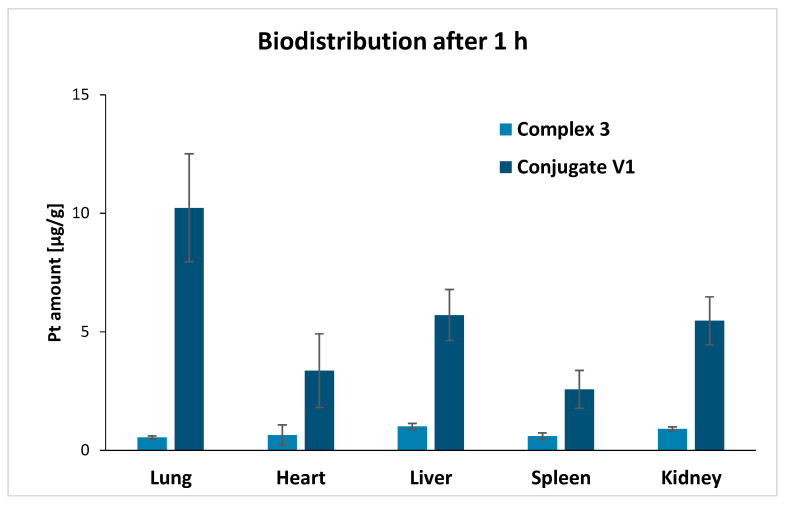
Comparison of the biodistribution investigation of platinum(IV) complex **3** and conjugate **V1** showing the accumulation of platinum in different organs 1 h after administration (complex 3: *n* = 5, conjugate V1: *n* = 4). The values are presented as mean ± SD. The data of complex **3** were published previously [19].

**Figure 2:**
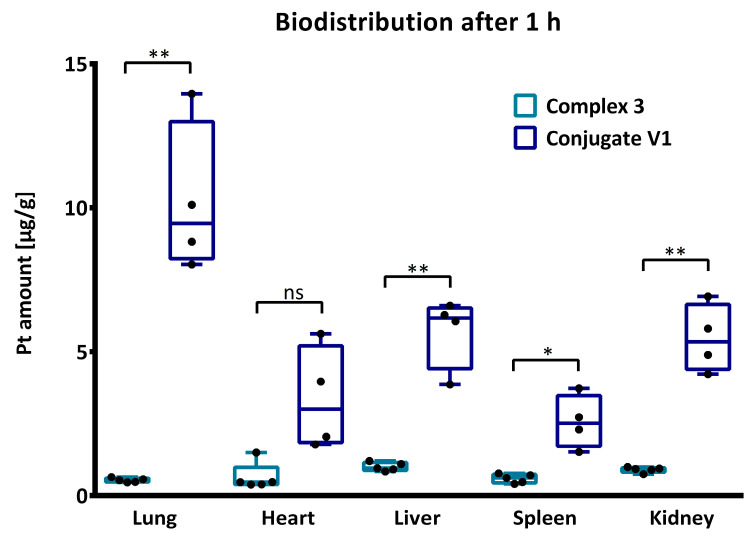
For the biodistribution, unpaired *t*-test with Welch’s correction was conducted for the determination of significances using following abbreviations: ns = not significant, * *p* < 0.05, ** *p* < 0.01. The values are presented as mean ± SEM.

In order to obtain a deeper understanding of the target organs for these novel conjugates, biodistribution studies were performed with oxaliplatin analogue **3** in comparison to its conjugate **V1**. Doses of 0.15 mg/100 µL of **3** and 0.95 mg/100 µL of **V1** equivalent to 5.5 mg/kg oxaliplatin were injected intravenously into healthy BALB/c mice. The administered concentration was well tolerated by the mice and did not reveal any signs of gross toxicity. One hour after administration, the major organs were collected and their platinum content was determined via ICP-MS (Figure 1 and Figure 2).

Complex **3** displayed a relatively equal distribution in lung, heart and liver, whereas the highest platinum level was detected in liver followed by kidneys similar to previously reported findings [33]. Contrary, the lung was the significantly preferred organ of conjugate **V1** followed by liver and kidney. Interestingly, the high accumulation in the lung was not observed in previous biodistribution studies of GCPQ polymers [34,35], enabling interesting opportunities for further investigations.

## 3. Materials and Methods

### 3.1. Materials

All solvents and chemicals were acquired from commercial suppliers and were used as received. The following chemicals were used for the synthesis: K_2_PtCl_4_ (Assay: 46.69% Pt) (Johnson Matthey, Zurich, Switzerland), glycol chitosan (Assay: 78.2%) (Wako, Osaka, Japan), palmitic acid *N*-hydroxysuccinimide (PNS) (99%) (Biosynth Carbosynth, Billingham, UK), *N*-methyl-2-pyrrolidon (NMP) (99%) (Fischer Scientific, Schwerte, Germany), sodium hydroxide (97%) (Sigma-Aldrich, St. Louis, MO, USA), sodium iodide (≥99%) (Acros Organics, Geel, Belgium), methyl iodide (99%) (Thermo Scientific, Budapest, Hungary), carbonyldiimidazole (CDI) (≥97%) (Sigma Aldrich, Steinheim, Germany), Amberlite^®^ IRA-410 chloride form (Sigma Aldrich, Saint-Quentin-Fallavier, France), *tert*-butyl methyl ether (>99.5%) (Fisher Scientific, Svhwerte, Germany), acetone (>99.5%) (Sigma-Aldrich, Saint-Quentin-Fallavier, France), DMSO (>99.8%) (Acros Organics, Geel, Belgium), triethylamine (99%) (Acros Organics, Geel, Belgium). Dialysis tubing (molecular weight cut off (MWCO) = 3.5 kDa) from Medicell Membranes (London, UK) was used for the dialysis of GCPQ polymers, whereas Trial Kit Spectra/Por^®^ 3 (MWCO = 3.5 kDa) dialysis tubing was used for all conjugates and was obtained from Carl Roth (Karlsruhe, Germany). Milli-Q water (18.2 MΩ cm, Milli-Q Advantage) was used for dialysis. Conjugation reactions containing platinum(IV) complexes were performed in darkness with glass coated magnetic stirring bars.

### 3.2. NMR Spectroscopy

NMR spectroscopy measurements were performed with a Bruker Avance NEO 500 MHz NMR spectrometer at 500.32 (^1^H) and 107.55 MHz (^195^Pt) in D_2_O at 298 K. ^1^H NMR spectra were measured relative to the solvent resonance of δ = 4.79 ppm, whereas K_2_[PtCl_4_] was used as external reference for ^195^Pt NMR spectra.

### 3.3. Inductively Coupled Plasma Mass Spectrometry (ICP-MS)

For digestion, the conjugates (0.5–1.5 mg) were dissolved in 2 mL of HNO_3_ (20%) and 0.1 mL of H_2_O_2_ (30%) and heated over a period of 6 h with a temperature-controlled heating plate of graphite from Labter. Dilutions of 1:10,000 were prepared with HNO_3_ (3%) for all samples, and afterwards, the total platinum amount was determined with an Agilent 7800 ICP-MS instrument using rhenium as internal reference. Ten replicates for each sample were measured and analysed with the Agilent MassHunter software package (Workstation Software, Version C.01.04, 2018, Agilent, Santa Clara, CA, USA).

### 3.4. Gel permeation Chromatography Coupled to Multiangle Laser Light Scattering Detector (GPC-MALLS)

The used mobile phase consisted of acetate buffer and methanol (65:35). The acetate buffer (pH = 4) was prepared by dissolving sodium acetate (3.05 g) in 800 mL of H_2_O, adjusting the pH with acetic acid (11 mL), and, finally, the volume was made up to 1 L with H_2_O. A primary sample was prepared by dissolving the GCPQ samples (25 mg) in mobile phase (2.5 mL) and filtering through a 0.2 µm PTFE syringe filter. For dn/dc determination, primary samples were diluted in mobile phase to concentrations of 0.1, 0.2, 0.3, 0.4, 0.5 and 0.6 mg/mL. Each dilution was then injected directly into the Optilab TrEX, allowing the change in refractive index (dRI) to settle between injections (≈30 s). The experiment was started and ended with an injection of mobile phase to be used as a baseline. For molecular weight determination, the primary sample without dilution (100 µL, 100 µg) was analysed using the mobile phase described above, at a flow rate of 0.7 mL/min, and separated using a PolySep-GFC-P 4000 column fitted with a PolySep-GFC-P guard column. The signal was detected using DAWN HELEOS II and Optilab TrEX detectors from Wyatt Technology (Cambridge, UK) and analysed using ASTRA software version 5.3.4.14.

### 3.5. Synthesis

#### 3.5.1. GCPQ Polymers

The degradation of glycol chitosan, as well as the palmitoylation and quaternisation, was adapted according to standard procedures [24]. The levels of palmitoylation and quaternisation were calculated by the comparison of the ratios of palmitoyl methyl protons, quaternary ammonium protons and sugar backbone protons, respectively, by means of ^1^H NMR spectroscopy.

##### General Procedure 1: Degradation and Palmitoylation of Glycol Chitosan

Glycol chitosan (MW ≈ 120 kDa) was heated with hydrochloric acid between 14 and 21 h resulting in degraded glycol chitosan (dGC). Afterwards, dGC and palmitic acid *N*-hydroxysuccinimide (PNS) were dissolved in a DMSO/trimethylamine (TEA) mixture and stirred overnight under the absence of light. The formed palmitoyl glycol chitosan (PGC) was precipitated by a mixture of acetone/*tert*-butyl methyl ether (1:2), filtered off and dried under reduced pressure.

1. Palmitoyl glycol chitosan (**P7GC**)

General procedure 1. dGC (15 h) (5.031 g), PNS (591 mg), DMSO + 3.7% TEA (155 mL). Yield: 1.664 g.

2. Palmitoyl glycol chitosan (**P8GC**)

General procedure 1. dGC (21 h) (2.500 g), PNS (328 mg), DMSO + 3.7% TEA (75 mL). Yield: 1.310 g.

3. Palmitoyl glycol chitosan (**P21aGC**)

General procedure 1. dGC (16 h) (5.001 g), PNS (1.760 g), DMSO + 3.7% TEA (155 mL). Yield: 3.084 g.

4. Palmitoyl glycol chitosan (**P21bGC**)

General procedure 1. dGC (15 h) (4.533 g), PNS (1.607 g), DMSO + 3.7% TEA (140 mL). Yield: 5.551 g.

5. Palmitoyl glycol chitosan (**P22GC**)

General procedure 1. dGC (14 h) (5.045 g), PNS (1.695 g), DMSO + 3.7% TEA (156 mL). Yield: 4.529 g.

##### General Procedure 2: Quaternisation of Palmitoyl Glycol Chitosan

PGC was dissolved in *N*-methyl-2-pyrrolidon (NMP) and sodium hydroxide, sodium iodide and methyl iodide were added under a nitrogen atmosphere. The reaction solution was stirred at 36 °C between 1 and 25 h. The crude GCPQ was precipitated by 0.1 M NaOH and collected via filtration. GCPQ was resuspended in methanol and dialysed (MWCO = 3.5 kDa) against distilled water over 48 h. Afterwards, the product solution was stirred with Amberlite IRA 410 ion exchange resin for approximately 20 min to remove iodide. Amberlite was filtered off and the product containing filtrate was adjusted to pH between 3 and 4 by adding concentrated HCl. Finally, GCPQ was obtained via freeze-drying.

1. Quaternary ammonium palmitoyl glycol chitosan **GCP7Q7**

General procedure 2. **P7GC** (1.664 g), NMP (97 mL), NaOH (225 mg), NaI (259 mg), ICH_3_ (2.5 mL), reaction time: 1 h. Yield: 1.434 g. ^1^H NMR (D_2_O): δ = 5.37 + 4.89–5.19 (m, O-C*H*-O, GCPQ backbone), 3.45–4.58 (m, GCPQ backbone), 3.21–3.37 (m, N(C*H*_3_)_3_, GCPQ), 3.01 + 2.81 (b, N(C*H*_3_)_2_ and NC*H*_3_, GCPQ), 2.24 (b, (C=O)C*H*_2_, palmitoyl), 1.94–2.07 (m, acetyl-C*H*_3_, GCPQ), 1.53 (b, (C=O)CH_2_C*H*_2_, palmitoyl), 1.20 (b, C*H*_2_, palmitoyl), 0.76–0.86 (m, C*H*_3_, palmitoyl) ppm.

2. Quaternary ammonium palmitoyl glycol chitosan **GCP8Q10**

General procedure 2. **P8GC** (1.310 g), NMP (104 mL), NaOH (169 mg), ICH_3_ (5.0 mL), reaction time: 1 h, precipitated with diethyl ether. Yield: 808 g. ^1^H NMR (MeOD + 5 µL DCl): δ = 5.40 + 4.70–5.15 (m, O-C*H*-O, GCPQ backbone), 3.50–4.68 (m, GCPQ backbone), 3.31–3.43 (m, N(C*H*_3_)_3_, GCPQ), 3.00–3.20 (m, N(C*H*_3_)_2_ and NC*H*_3_, GCPQ), 2.30 (b, (C=O)C*H*_2_, palmitoyl), 2.00–2.12 (m, acetyl-C*H*_3_, GCPQ), 1.63 (b, (C=O)CH_2_C*H*_2_, palmitoyl), 1.25 (b, C*H*_2_, palmitoyl), 0.85–0.91 (m, C*H*_3_, palmitoyl) ppm.

3. Quaternary ammonium palmitoyl glycol chitosan **GCP21Q12**

General procedure 2. **P21aGC** (3.084 g), NMP (179 mL), NaOH (417 mg), NaI (476 mg), ICH_3_ (4.6 mL), reaction time: 3 h. Yield: 1.062 g. ^1^H NMR (MeOD + 5µL DCl): δ = 5.13–5.45 (m, O-C*H*-O, GCPQ backbone), 3.54–4.75 (m, GCPQ backbone), 3.40 (b, N(C*H*_3_)_3_, GCPQ), 3.14 (b, N(C*H*_3_)_2_ and NC*H*_3_, GCPQ), 2.30 (b, (C=O)C*H*_2_, palmitoyl), 2.04–2.14 (m, acetyl-C*H*_3_, GCPQ), 1.66 (b, (C=O)CH_2_C*H*_2_, palmitoyl), 1.33 (b, C*H*_2_, palmitoyl), 0.92 (t, ^3^J (^1^H, ^1^H) = 7.1 Hz, C*H*_3_, palmitoyl) ppm.

4. Quaternary ammonium palmitoyl glycol chitosan **GCP21Q27**

General procedure 2. **P21bGC** (5.551 g), NMP (320 mL), NaOH (750 mg), NaI (855 mg), ICH_3_ (8.3 mL), reaction time: 4 h. Yield: 3.333 g. ^1^H NMR (D_2_O): δ = 5.39 + 5.13 (b, O-C*H*-O, GCPQ backbone), 3.46- 4.42 (m, GCPQ backbone), 3.30 (b, N(C*H*_3_)_3_, GCPQ), 3.03 (b, N(C*H*_3_)_2_ and NC*H*_3_, GCPQ), 2.26 (b, (C=O)C*H*_2_, palmitoyl), 1.98–2.07 (m, acetyl-C*H*_3_, GCPQ), 1.56 (b, (C=O)CH_2_C*H*_2_, palmitoyl), 1.25 (b, C*H*_2_, palmitoyl), 0.86 (b, C*H*_3_, palmitoyl) ppm.

5. Quaternary ammonium palmitoyl glycol chitosan **GCP22Q33**

General procedure 2. **P22GC** (4.529 g), NMP (263 mL), NaOH (611 mg), NaI (701 mg), ICH_3_ (18.0 mL), reaction time: 25 h. Yield: 2.600 g. ^1^H NMR (D_2_O): δ = 5.37 + 5.08 (b, O-C*H*-O, GCPQ backbone), 3.44–4.46 (m, GCPQ backbone), 3.28 (b, N(C*H*_3_)_3_, GCPQ), 3.01 (b, N(C*H*_3_)_2_ and NC*H*_3_, GCPQ), 2.25 (b, (C=O)C*H*_2_, palmitoyl), 1.94–2.06 (m, acetyl-C*H*_3_, GCPQ), 1.53 (b, (C=O)CH_2_C*H*_2_, palmitoyl), 1.22 (b, C*H*_2_, palmitoyl), 0.82 (b, C*H*_3_, palmitoyl) ppm.

#### 3.5.2. Platinum(IV)–GCPQ Conjugates

##### General Procedure 3: Conjugation of Platinum(IV) Complexes to GCPQ

Synthesis of platinum(IV) complexes **1**–**3** as well as conjugation to the GCPQ polymer was performed according to a procedure published previously [19]. Platinum(IV) complex **1**, **2** or **3** dissolved in DMSO was treated with CDI and stirred for half an hour. In the meantime, a solution of GCPQ dissolved in DMSO and trimethylamine (TEA) was prepared and added to the solution containing the platinum(IV) complex. The mixture was stirred overnight at room temperature. Purification was performed via dialysis against Milli-Q water using dialysis tubing with a MWCO = 3.5 kDa. After the adjustment of a pH of 3 with HCl, the final conjugates were obtained by lyophilisation.

1. Complex **1** coupled to **GCP7Q7** (**C1**)

General procedure 3. **1** (25 mg, 0.05 mmol), CDI (21 mg, 0.13 mmol), **GCP7Q7** (72 mg, 0.0055 mmol), TEA (29 µL, 0.21 mmol). Yield: 65 mg. ICP-MS (Pt): 50.7 g/kg. ^1^H NMR (D_2_O): δ = 5.10 (b, O-C*H*-O, GCPQ backbone), 3.60–4.43 (m, GCPQ backbone), 3.30–3.44 (m, N(C*H*_3_)_3_, GCPQ), 3.09 + 2.89 (b, N(C*H*_3_)_2_ and NC*H*_3_, GCPQ), 2.68–2.72 (m, C*H*_2_, succinato), 2.62- 2.66 (m, C*H*_2_, succinato), 2.32 (b, (C=O)C*H*_2_, palmitoyl), 2.05–2.16 (m, amide-C*H*_3_, GCPQ), 2.12 (b, C*H*_3_, acetato), 1.62 (b, (C=O)CH_2_C*H*_2_, palmitoyl), 1.29 (b, C*H*_2_, palmitoyl), 0.85–0.97 (m, C*H*_3_, palmitoyl) ppm. ^195^Pt NMR (D_2_O): δ = 2705 ppm.

2. Complex **1** coupled to **GCP21Q12** (**C2**)

General procedure 3. **1** (20 mg, 0.04 mmol), CDI (17 mg, 0.11 mmol), **GCP21Q12** (73 mg, 0.0061 mmol), TEA (23 µL, 0.17 mmol). Yield: 65 mg. ICP-MS (Pt): 37.1 g/kg. ^1^H NMR (D_2_O): δ = 5.14 (b, O-C*H*-O, GCPQ backbone), 3.52–4.53 (m, GCPQ backbone), 3.36 (b, N(C*H*_3_)_3_, GCPQ), 3.09 (b, N(C*H*_3_)_2_ and NC*H*_3_, GCPQ), 2.67–2.72 (m, C*H*_2_, succinato)(in part overlapping with DMSO signal), 2.61–2.66 (m, C*H*_2_, succinato), 2.32 (b, (C=O)C*H*_2_, palmitoyl), 2.09–2.15 (m, amide-C*H*_3_, GCPQ), 2.12 (b, C*H*_3_, acetato), 1.62 (b, (C=O)CH_2_C*H*_2_, palmitoyl), 1.32 (b, C*H*_2_, palmitoyl), 0.92 (b, C*H*_3_, palmitoyl) ppm. ^195^Pt NMR (D_2_O): δ = 2705 ppm.

3. Complex **1** coupled to **GCP21Q27** (**C3**)

General procedure 3. **1** (15 mg, 0.03 mmol), CDI (13 mg, 0.08 mmol), **GCP21Q27** (79 mg, 0.0068 mmol), TEA (17 µL, 0.13 mmol). Yield: 72 mg. ICP-MS (Pt): 34.9 g/kg. ^1^H NMR (D_2_O): δ = 5.43 + 5.15 (b, O-C*H*-O, GCPQ backbone), 3.51–4.56 (m, GCPQ backbone), 3.36 (b, N(C*H*_3_)_3_, GCPQ), 3.08 (b, N(C*H*_3_)_2_ and NC*H*_3_, GCPQ), 2.67–2.72 (m, C*H*_2_, succinato), 2.61–2.65 (m, C*H*_2_, succinato), 2.32 (b, (C=O)C*H*_2_, palmitoyl), 2.05–2.16 (m, amide-C*H*_3_, GCPQ), 2.12 (b, C*H*_3_, acetato), 1.63 (b, (C=O)CH_2_C*H*_2_, palmitoyl), 1.33 (b, C*H*_2_, palmitoyl), 0.93 (b, C*H*_3_, palmitoyl) ppm. ^195^Pt NMR (D_2_O): δ = 2705 ppm.

4. Complex **1** coupled to **GCP22Q33** (**C4**)

General procedure 3. **1** (15 mg, 0.03 mmol), CDI (13 mg, 0.08 mmol), **GCP22Q33** (61 mg, 0.0034 mmol), TEA (17 µL, 0.13 mmol). Yield: 52 mg. ICP-MS (Pt): 50.9 g/kg. ^1^H NMR (D_2_O): δ = 5.45 + 5.13 (b, O-C*H*-O, GCPQ backbone), 3.51–4.54 (m, GCPQ backbone), 3.37 (b, N(C*H*_3_)_3_, GCPQ), 3.09 (b, N(C*H*_3_)_2_ and NC*H*_3_, GCPQ), 2.68–2.72 (m, C*H*_2_, succinato), 2.62–2.66 (m, C*H*_2_, succinato), 2.33 (b, (C=O)C*H*_2_, palmitoyl), 2.07–2.15 (m, amide-C*H*_3_, GCPQ), 2.12 (b, C*H*_3_, acetato), 1.63 (b, (C=O)CH_2_C*H*_2_, palmitoyl), 1.32 (b, C*H*_2_, palmitoyl), 0.93 (b, C*H*_3_, palmitoyl) ppm. ^195^Pt NMR (D_2_O): δ = 2705 ppm.

5. Complex **2** coupled to **GCP21Q12** (**C5**)

General procedure 3. **2** (20 mg, 0.04 mmol), CDI (15 mg, 0.09 mmol), **GCP21Q12** (64 mg, 0.0054 mmol), TEA (21 µL, 0.15 mmol). Yield: 58 mg. ICP-MS (Pt): 62.3 g/kg. ^1^H NMR (D_2_O): δ = 5.45 + 5.18 (b, O-C*H*-O, GCPQ backbone), 3.61–4.42 (m, GCPQ backbone), 3.36 (b, N(C*H*_3_)_3_, GCPQ), 3.09 (b, N(C*H*_3_)_2_ and NC*H*_3_, GCPQ), 2.63–2.71 (m, C*H*_2_, succinato; C-C*H*_2_, cyclobutyl), 2.57–2.62 (m, C*H*_2_, succinato), 2.32 (b, (C=O)C*H*_2_, palmitoyl), 2.06–2.10 (m, amide-C*H*_3_, GCPQ), 2.07 (b, C*H*_3_, acetato), 2.03 (p, ^3^J (^1^H, ^1^H = 8.2 Hz), C*H*_2_, cyclobutyl), 1.62 (b, (C=O)CH_2_C*H*_2_, palmitoyl), 1.30 (b, C*H*_2_, palmitoyl), 0.90 (b, C*H*_3_, palmitoyl) ppm. ^195^Pt NMR (D_2_O): δ = 3509 ppm.

6. Complex **2** coupled to **GCP21Q27** (**C6**)

General procedure 3. **2** (50 mg, 0.09 mmol), CDI (37 mg, 0.23 mmol), **GCP21Q27** (114 mg, 0.0099 mmol), TEA (51 µL, 0.37 mmol). Yield: 106 mg. ICP-MS (Pt): 25.4 g/kg. ^1^H NMR (D_2_O): δ = 5.43 + 5.16 (b, O-C*H*-O, GCPQ backbone), 3.50–4.51 (m, GCPQ backbone), 3.36 (b, N(C*H*_3_)_3_, GCPQ), 3.09 (b, N(C*H*_3_)_2_ and NC*H*_3_, GCPQ), 2.63–2.71 (m, C*H*_2_, succinato; C-C*H*_2_, cyclobutyl), 2.57–2.62 (m, C*H*_2_, succinato), 2.32 (b, (C=O)C*H*_2_, palmitoyl), 2.05–2.13 (m, amide-C*H*_3_, GCPQ), 2.07 (b, C*H*_3_, acetato), 2.03 (p, ^3^J (^1^H, ^1^H = 8.2 Hz), C*H*_2_, cyclobutyl), 1.62 (b, (C=O)CH_2_C*H*_2_, palmitoyl), 1.30 (b, C*H*_2_, palmitoyl), 0.90 (b, C*H*_3_, palmitoyl) ppm. ^195^Pt NMR (D_2_O): δ = 3509 ppm.

7. Complex **2** coupled to **GCP22Q33** (**C7**)

General procedure 3. **2** (35 mg, 0.06 mmol), CDI (26 mg, 0.16 mmol), **GCP22Q33** (62 mg, 0.0034 mmol), TEA (35 µL, 0.26 mmol). Yield: 50 mg. ICP-MS (Pt): 66.1 g/kg. ^1^H NMR (D_2_O): δ = 5.42 + 5.15 (b, O-C*H*-O, GCPQ backbone), 3.49–4.43 (m, GCPQ backbone), 3.34 (b, N(C*H*_3_)_3_, GCPQ), 3.08 (b, N(C*H*_3_)_2_ and NC*H*_3_, GCPQ), 2.61–2.69 (m, C*H*_2_, succinato; C-C*H*_2_, cyclobutyl), 2.55–2.60 (m, C*H*_2_, succinato), 2.30 (b, (C=O)C*H*_2_, palmitoyl), 2.03–2.11 (m, amide-C*H*_3_, GCPQ), 2.05 (b, C*H*_3_, acetato), 2.00 (p, ^3^J (^1^H, ^1^H = 8.0 Hz), C*H*_2_, cyclobutyl), 1.60 (b, (C=O)CH_2_C*H*_2_, palmitoyl), 1.28 (b, C*H*_2_, palmitoyl), 0.89 (b, C*H*_3_, palmitoyl) ppm. ^195^Pt NMR (D_2_O): δ = 3509 ppm.

8. Complex **3** coupled to **GCP7Q7** (**C8**)

General procedure 3. **3** (202 mg, 0.35 mmol), CDI (141 mg, 0.87 mmol), **GCP7Q7** (158 mg, 0.0121 mmol), TEA (193 µL, 1.40 mmol). Yield: 129 mg. ICP-MS (Pt): 23.6 g/kg. ^1^H NMR (D_2_O): δ = 4.91–5.22 (m, O-C*H*-O, GCPQ backbone), 3.44–4.44 (m, GCPQ backbone), 3.24–3.40 (m, N(C*H*_3_)_3_, GCPQ), 2.92–3.20 (m, C*H*, DACH), 2.82 (m, N(C*H*_3_)_2_ and NC*H*_3_, GCPQ), 2.52–2.68 (m, C*H*_2_, succinato), 1.98–2.06 (m, C*H*_2_, DACH; (C=O)C*H*_2_, palmitoyl), 2.03 (b, C*H*_3_, acetato; amide-C*H*_3_, GCPQ), 1.47–1.63 (m, C*H*_2_, DACH; (C=O)CH_2_C*H*_2_, palmitoyl), 1.23 (b, C*H*_2_, palmitoyl; C*H*_2_, DACH), 0.78–0.89 (m, C*H*_3_, palmitoyl) ppm. ^195^Pt NMR (D_2_O): δ = 3213 ppm.

9. Complex **3** coupled to **GCP21Q12** (**C9**)

General procedure 3. **3** (35 mg, 0.06 mmol), CDI (25 mg, 0.15 mmol), **GCP21Q12** (53 mg, 0.0044 mmol), TEA (34 µL, 0.24 mmol). Yield: 42 mg. ICP-MS (Pt): 118.5 g/kg. ^1^H NMR (D_2_O): δ = 3.53–4.36 (m, GCPQ backbone), 3.34 (b, N(C*H*_3_)_3_, GCPQ), 2.84–3.03 (m, C*H*, DACH), 2.49–2.72 (m, C*H*_2_, succinato; N(C*H*_3_)_2_ and NC*H*_3_, GCPQ), 2.35–2.45 (m, C*H*_2_, succinato), 2.24–2.35 (m, C*H*_2_, DACH; (C=O)C*H*_2_, palmitoyl), 2.07 (b, C*H*_3_, acetato; amide-C*H*_3_, GCPQ), 1.51–1.74 (m, C*H*_2_, DACH; (C=O)CH_2_C*H*_2_, palmitoyl), 1.31 (b, C*H*_2_, palmitoyl; C*H*_2_, DACH), 0.83–1.00 (m, C*H*_3_, palmitoyl) ppm. ^195^Pt NMR (D_2_O): δ = 3223 ppm.

10. Complex **3** coupled to **GCP21Q27** (**C10**)

General procedure 3. **3** (30 mg, 0.05 mmol), CDI (21 mg, 0.13 mmol), **GCP21Q27** (65 mg, 0.0056 mmol), TEA (29 µL, 0.21 mmol). Yield: 57 mg. ICP-MS (Pt): 91.6 g/kg. ^1^H NMR (D_2_O): δ = 5.46 (b, O-C*H*-O, GCPQ backbone), 3.54–4.42 (m, GCPQ backbone), 3.35 (b, N(C*H*_3_)_3_, GCPQ), 2.84–3.01 (m, C*H*, DACH), 2.55–2.66 (m, C*H*_2_, succinato), 2.51 (b, N(C*H*_3_)_2_ and NC*H*_3_, GCPQ), 2.36–2.45 (m, C*H*_2_, succinato), 2.24–2.35 (m, C*H*_2_, DACH; (C=O)C*H*_2_, palmitoyl), 2.04–2.14 (m, amide-C*H*_3_, GCPQ), 2.07 (b, C*H*_3_, acetato), 1.52–1.73 (m, C*H*_2_, DACH; (C=O)CH_2_C*H*_2_, palmitoyl), 1.33 (b, C*H*_2_, palmitoyl; C*H*_2_, DACH), 0.93 (b, C*H*_3_, palmitoyl) ppm. ^195^Pt NMR (D_2_O): δ = 3223 ppm.

11. Complex **3** coupled to **GCP22Q33** (**C11**)

General procedure 3. **3** (29 mg, 0.05 mmol), CDI (18 mg, 0.11 mmol), **GCP22Q33** (51 mg, 0.0028 mmol), TEA (24 µL, 0.17 mmol). Yield: 41 mg. ICP-MS (Pt): 88.7 g/kg. ^1^H NMR (D_2_O): δ = 5.44 (b, O-C*H*-O, GCPQ backbone), 3.49–4.43 (m, GCPQ backbone), 3.34 (b, N(C*H*_3_)_3_, GCPQ), 2.84–3.01 (m, C*H*, DACH), 2.55–2.65 (m, C*H*_2_, succinato), 2.51 (b, N(C*H*_3_)_2_ and NC*H*_3_, GCPQ), 2.36–2.43 (m, C*H*_2_, succinato), 2.25–2.34 (m, C*H*_2_, DACH; (C=O)C*H*_2_, palmitoyl), 2.04–2.13 (m, amide-C*H*_3_, GCPQ), 2.07 (b, C*H*_3_, acetato), 1.53–1.73 (m, C*H*_2_, DACH; (C=O)CH_2_C*H*_2_, palmitoyl), 1.32 (b, C*H*_2_, palmitoyl; C*H*_2_, DACH), 0.92 (b, C*H*_3_, palmitoyl) ppm. ^195^Pt NMR (D_2_O): δ = 3223 ppm.

12. Complex **3** coupled to **GC8P10** (**V1**)

General procedure 3. **3** (78 mg, 0.14 mmol), CDI (46 mg, 0.29 mmol), **GCP8Q10** (74 mg, 0.0077 mmol), TEA (38 µL, 0.27 mmol). Yield: 25 mg. ICP-MS (Pt): 28.5 g/kg. ^1^H NMR (MeOD): δ = 5.21 (b, O-C*H*-O, GCPQ backbone), 3.54–4.32 (m, GCPQ backbone), 3.37 (b, N(C*H*_3_)_3_, GCPQ), 2.86–3.14 (m, N(C*H*_3_)_2_ and NC*H*_3_, GCPQ; C*H*, DACH), 2.52–2.74 (m, C*H*_2_, succinato), 2.19–2.37 (m, C*H*_2_, DACH; (C=O)C*H*_2_, palmitoyl), 1.98–2.08 (m, amide-C*H*_3_, GCPQ), 1.99 (b, C*H*_3_, acetato), 1.56–1.74 (m, C*H*_2_, DACH; (C=O)CH_2_C*H*_2_, palmitoyl), 1.32 (b, C*H*_2_, palmitoyl; C*H*_2_, DACH), 0.90 (t, ^3^J (^1^H, ^1^H) = 7.2 Hz, C*H*_3_, palmitoyl) ppm.

### 3.6. Cytotoxicity Tests

The MTT assay (96 h exposure time) in the human cancer cell lines SW480 (colon carcinoma), CH1/PA-1 (ovarian teratocarcinoma) and A549 (non-small-cell lung cancer) was performed as described in [32], with the exception that conjugates were dissolved either in supplemented MEM or sterile water and then serially diluted in the former medium. These cell lines were chosen to represent three malignancies that are clinical indications for platinum drugs on the one hand but possess different chemosensitivity profiles on the other, with CH1/PA-1 cells being broadly sensitive, SW480 (expressing P-glycoprotein) intermediately sensitive and A549 (expressing ABC transporters other than P-glycoprotein) multidrug-resistant, reflecting a different demand for improved platinum-based therapies in these malignancies. A description of the conducted MTT assay in the cancer cell line 4T1 (mammary carcinoma, 24 h exposure time) can be found in [19].

### 3.7. Biodistribution Studies

The Animal Welfare and Ethical Review Body at University College London (UCL) approved all animal experiments performed according to the Home Office Animals Scientific Procedures Act, 1986, United Kingdom. Non-tumour-bearing female BALB/c mice (20–22.5 g) (Harlan, UK) were used for the biodistribution of the oxaliplatin-based GCPQ conjugate and the free platinum(IV) complex **3**. Four to five mice per group were treated with doses of 0.15 mg/100 µL of complex **3** and 0.95 mg/100 µL of conjugate **V1** equivalent to 5.5 mg/kg oxaliplatin. The compounds were injected intravenously into the tail vein (100 µL/20 g body weight). After 1 h, heart, spleen, lung, liver and kidneys were removed, stored under liquid nitrogen and their platinum amount was detected via ICP-MS at the Institute of Inorganic Chemistry of the University of Vienna. For statistical analysis the *t*-test with Welsh’s correction was implemented in GraphPad Prism software (Version 6.01). Testing for normal distribution was not possible due to the small sample number (n = 4–5). The values are presented as mean ± SEM.

## 4. Conclusions

The conjugation of platinum(IV) analogues of cisplatin, carboplatin and oxaliplatin to GCPQ polymers differing in levels of palmitoylation and quaternisation led to a series of twelve novel conjugates with IC_50_ values in the low micromolar to nanomolar range. Remarkably, the conjugates featured higher antiproliferative activity compared to the respective platinum(IV) complex and most of the conjugates even outperformed the cytotoxicity of their corresponding platinum(II) precursors. Notably, increased cytotoxicity of factors up to 11 and 159 compared to platinum(II) and (IV) complexes, respectively, could additionally be observed in the multidrug-resistant non-small-cell lung cancer cell line A549. As a next step, investigations in other cancer cell lines, especially platinum(II)-resistant ones, could further disclose the full potential of platinum(IV)-based GCPQ-conjugates. Furthermore, a biodistribution study in non-tumour-bearing BALB/c mice was conducted with an oxaliplatin-based GCP8Q10 conjugate. The increased accumulation of the conjugate in the lung in comparison to the unloaded platinum(IV) complex combined with increased cytotoxicity in non-small-cell lung cancer cell line A549 revealed promising results for further activity experiments, especially with respect to different lung cancer types and lung metastases. In order to determine the potential of platinum(IV)-based GCPQ conjugates as a favourable new cancer treatment approach, additional investigations of their stability as well as pharmacokinetics and pharmacodynamics could provide clarification of therapeutic benefits and potential side effects. Finally, studies of GCPQ as a drug delivery system for platinum(IV) complexes or combined with other anticancer agents (e.g., doxorubicin) could further result in promising approaches for future cancer treatments.

## Figures and Tables

**Table 1:** Summary of parameters of the five synthesised GCPQ polymers.

Sample	Mol % Palmitoylation	Mol % Quaternisation	QPR *	MW [kDa]
**GCP7Q7**	7	7	1.0	13.1
**GCP8Q10**	8	10	1.3	9.6
**GCP21Q12**	21	12	0.6	12.0
**GCP21Q27**	21	27	1.3	11.5
**GCP22Q33**	22	33	1.5	18.0

The following nomenclature is used for the GCPQ polymers: GCPxQy with x = mol % of palmitoylation and y = mol % of quaternisation. * QPR = (mol % quaternisation)/(mol % palmitoylation), parameter for hydrophilicity.

**Table 2:** Overview of the newly developed platinum(IV)-containing conjugates **C1**–**C11**, **V1**.

Sample	Pt(IV)	GCPQ	Pt(IV) Units Per GCPQ	MW [kDa]
**C1**	**1**	**GCP7Q7**	3.9	14.9
**C2**	**1**	**GCP21Q12**	2.5	13.1
**C3**	**1**	**GCP21Q27**	2.3	12.6
**C4**	**1**	**GCP22Q33**	5.3	20.5
**C5**	**2**	**GCP21Q12**	4.6	14.4
**C6**	**2**	**GCP21Q27**	1.6	12.4
**C7**	**2**	**GCP22Q33**	7.5	22.0
**C8**	**3**	**GCP7Q7**	1.7	14.0
**C9**	**3**	**GCP21Q12**	11.0	18.1
**C10**	**3**	**GCP21Q27**	7.3	15.6
**C11**	**3**	**GCP22Q33**	11.0	24.1
**V1**	**3**	**GCP8Q10**	1.5	10.4

**Table 3:** IC_50_ values (means ± standard deviations from at least three independent experiments) of conjugates **C1**–**C11** as well as their corresponding platinum(II) and platinum(IV) complexes in the three human cancer cell lines according to the MTT assay (96 h exposure).

Sample	Pt(IV)	GCPQ	Pt(IV) Units Per Polymer	IC_50_ [µM] A549	IC_50_ [µM] CH1/PA-1	IC_50_ [µM] SW480
cisplatin [32]	-	-	-	3.8 ± 1.0	0.073 ± 0.001	2.3 ± 0.2
carboplatin [32]	-	-	-	38 ± 3	0.79 ± 0.11	42 ± 10
oxaliplatin [32]	-	-	-	0.98 ± 0.21	0.18 ± 0.01	0.29 ± 0.05
**1** [19]	**1**	-	-	99 ± 17	1.2 ± 0.5	47 ± 10
**2** [19]	**2**	-	-	>200	16 ± 6	>200
**3** [19]	**3**	-	-	70 ± 29	4.1 ± 0.6	22 ± 8
**GCP7Q7**	-	**GCP7Q7**	-	>12.5	2.4 ± 0.4	5.0 ± 0.6
GCP21Q12 *	-	**GCP21Q12**	-	-	-	-
**GCPQ21Q27**	-	**GCPQ21Q27**	-	2.2 ± 0.5	1.6 ± 0.1	1.1 ± 0.1
**GCP22Q33**	-	**GCP22Q33**	-	1.8 ± 0.2	0.95 ± 0.13	0.83 ± 0.08
C1 **	**1**	**GCP7Q7**	3.9	-	-	-
**C2**	**1**	**GCP21Q12**	2.5	8.7 ± 2.0	0.015 ± 0.005	1.1 ± 0.2
**C3**	**1**	**GCP21Q27**	2.3	1.3 ± 0.4	0.0046 ± 0.0015	0.12 ± 0.03
**C4**	**1**	**GCP22Q33**	5.3	1.5 ± 0.4	0.0025 ± 0.0005	0.20 ± 0.04
**C5**	**2**	**GCP21Q12**	4.6	>12.5	0.25 ± 0.09	1.3 ± 0.1
**C6**	**2**	**GCP21Q27**	1.6	2.3 ± 0.9	0.028 ± 0.007	1.3 ± 0.2
**C7**	**2**	**GCP22Q33**	7.5	2.0 ± 0.3	0.018 ± 0.002	0.64 ± 0.13
**C8**	**3**	**GCP7Q7**	1.7	2.0 ± 0.9	0.040 ± 0.012	0.21 ± 0.03
**C9**	**3**	**GCP21Q12**	11.0	0.15 ± 0.07	0.0058 ± 0.0016	0.014 ± 0.004
**C10**	**3**	**GCP21Q27**	7.3	0.10 ± 0.03	0.0036 ± 0.0014	0.011 ± 0.002
**C11**	**3**	**GCP22Q33**	11.0	0.040 ± 0.011	0.0018 ± 0.0005	0.0070 ± 0.0013

* Could not be determined due to precipitation in the test medium. ** Could not be determined due to insufficient solubility.

**Table 4:** Factors of increase (decrease) in cytotoxicity of conjugates **C2**–**C11** in human cancer cell lines compared to corresponding platinum(II) and (IV) complexes by taking into account the respective number of platinum(IV) units per GCPQ polymer of the conjugates.

	Cytotoxicity Factors Compared to Pt(II)			Cytotoxicity Factors Compared to Pt(IV)		
Sample	A549	CH1/PA-1	SW480	A549	CH1/PA-1	SW480
**C2**	0.17	1.9	0.81	4.5	32	17
**C3**	1.3	7.4	8.3	33	121	170
**C4**	0.48	6.0	2.2	13	98	44
**C5**	-	0.69	7.1	-	14	34
**C6**	11	18	20	55	357	97
**C7**	2.5	5.9	8.8	13	119	42
**C8**	0.29	2.6	0.81	29	60	62
**C9**	0.64	2.8	2.0	46	64	150
**C10**	1.3	6.8	3.5	96	156	265
**C11**	2.2	9.1	3.8	159	207	286

**Table 5:** IC_50_ value (means ± standard error) of conjugate **V1** in the murine mammary carcinoma cell line 4T1. The IC_50_ value was obtained from 2 independent MTT assays.

Sample	Pt(IV)	GCPQ	Pt(IV) Units Per GCPQ	IC_50_ [µM] 4T1
**V1**	**3**	**GCP8Q10**	1.51	0.82 ± 0.08

## Data Availability

The data presented in this study are available in the article and in the Supplementary Materials.

## Supplementary Materials

The following supporting information can be downloaded at: https://www.mdpi.com/article/10.3390/ph16071027/s1, Figure S1: ^1^H NMR spectrum of polymer **GCP21Q12**; Figure S2: ^1^H NMR spectrum of conjugate **C2**; Figure S3: ^195^Pt NMR spectrum of conjugate **C2**; Figure S4: ^1^H NMR spectrum of conjugate **C6**; Figure S5: ^195^Pt NMR spectrum of conjugate **C6**; Figure S6: ^1^H NMR spectrum of conjugate **C11**; Figure S7: ^195^Pt NMR spectrum of conjugate **C11**; Figure S8: ^1^H NMR spectra of **3**, **C11** and **GCP22Q33**; Figure S9: Concentration–effect curves of **GCP7Q7** and **C8** in A549, CH1/PA-1 and SW480 cells; Figure S10: Concentration–effect curves of **GCPQ21Q27**, **C3**, **C6** and **C10** in A549, CH1/PA-1 and SW480 cells; Figure S11: Concentration–effect curves of **GCP22Q33**, **C4**, **C7** and **C11** in A549, CH1/PA-1 and SW480 cells; Figure S12: Concentration–effect curves of **C2**, **C5** and **C9** in A549, CH1/PA-1 and SW480 cells; Table S1: Overview of the water solubility of conjugates **C1**–**C11**.

## References

[1] Živković M.D., Kljun J., Ilic-Tomic T., Pavic A., Veselinović A., Manojlović D.D., Nikodinovic-Runic J., Turel I. (2018). A New Class of Platinum(II) Complexes with the Phosphine Ligand Pta Which Show Potent Anticancer Activity. Inorg. Chem. Front..

[2] Ramu V., Gill M.R., Jarman P.J., Turton D., Thomas J.A., Das A., Smythe C. (2015). A Cytostatic Ruthenium(II)-Platinum(II) Bis(Terpyridyl) Anticancer Complex That Blocks Entry into S Phase by up-Regulating P27KIP1. Chem. A Eur. J..

[3] Ghosh S. (2019). Cisplatin: The First Metal Based Anticancer Drug. Bioorg. Chem..

[4] Oun R., Moussa Y.E., Wheate N.J. (2018). The Side Effects of Platinum-Based Chemotherapy Drugs: A Review for Chemists. Dalton Trans..

[5] Deo K.M., Ang D.L., McGhie B., Rajamanickam A., Dhiman A., Khoury A., Holland J., Bjelosevic A., Pages B., Gordon C. (2018). Platinum Coordination Compounds with Potent Anticancer Activity. Coord. Chem. Rev..

[6] Gibson D. (2021). Platinum(IV) Anticancer Agents; Are We En Route to the Holy Grail or to a Dead End?. J. Inorg. Biochem..

[7] Marotta C., Giorgi E., Binacchi F., Cirri D., Gabbiani C., Pratesi A. (2023). An Overview of Recent Advancements in Anticancer Pt(IV) Prodrugs: New Smart Drug Combinations, Activation and Delivery Strategies. Inorg. Chim. Acta.

[8] Wexselblatt E., Gibson D. (2012). What Do We Know about the Reduction of Pt(IV) pro-Drugs?. J. Inorg. Biochem..

[9] Xu Z., Wang Z., Deng Z., Zhu G. (2021). Recent Advances in the Synthesis, Stability, and Activation of Platinum(IV) Anticancer Prodrugs. Coord. Chem. Rev..

[10] Gibson D. (2016). Platinum(IV) Anticancer Prodrugs-Hypotheses and Facts. Dalton Trans..

[11] Ritacco I., Mazzone G., Russo N., Sicilia E. (2016). Investigation of the Inertness to Hydrolysis of Platinum(IV) Prodrugs. Inorg. Chem..

[12] Kenny R.G., Chuah S.W., Crawford A., Marmion C.J. (2017). Platinum(IV) Prodrugs—A Step Closer to Ehrlich’s Vision?. Eur. J. Inorg. Chem..

[13] Hall M.D., Mellor H.R., Callaghan R., Hambley T.W. (2007). Basis for Design and Development of Platinum(IV) Anticancer Complexes. J. Med. Chem..

[14] Han X., Sun J., Wang Y., He Z. (2015). Recent Advances in Platinum (IV) Complex-Based Delivery Systems to Improve Platinum (II) Anticancer Therapy. Med. Res. Rev..

[15] Zhong Y., Jia C., Zhang X., Liao X., Yang B., Cong Y., Pu S., Gao C. (2020). Targeting Drug Delivery System for Platinum(IV)-Based Antitumor Complexes. Eur. J. Med. Chem..

[16] Jia C., Deacon G.B., Zhang Y., Gao C. (2021). Platinum(IV) Antitumor Complexes and Their Nano-Drug Delivery. Coord. Chem. Rev..

[17] Subhan M.A., Yalamarty S.S.K., Filipczak N., Parveen F., Torchilin V.P. (2021). Recent Advances in Tumor Targeting via Epr Effect for Cancer Treatment. J. Pers. Med..

[18] Shi Y., van der Meel R., Chen X., Lammers T. (2020). The EPR Effect and beyond: Strategies to Improve Tumor Targeting and Cancer Nanomedicine Treatment Efficacy. Theranostics.

[19] Lerchbammer-Kreith Y., Sommerfeld N.S., Cseh K., Weng-Jiang X., Odunze U., Schätzlein A.G., Uchegbu I.F., Galanski M.S., Jakupec M.A., Keppler B.K. (2023). Platinum(IV)-Loaded Degraded Glycol Chitosan as Efficient Platinum(IV) Drug Delivery Platform. Pharmaceutics.

[20] Shukla S.K., Mishra A.K., Arotiba O.A., Mamba B.B. (2013). Chitosan-Based Nanomaterials: A State-of-the-Art Review. Int. J. Biol. Macromol..

[21] Martau G.A., Mihai M., Vodnar D.C. (2019). The Use of Chitosan, Alginate, and Pectin in the Biomedical and Food Sector-Biocompatibility, Bioadhesiveness, and Biodegradability. Polymers.

[22] Trapani A., Sitterberg J., Bakowsky U., Kissel T. (2009). The Potential of Glycol Chitosan Nanoparticles as Carrier for Low Water Soluble Drugs. Int. J. Pharm..

[23] Ryu J.H., Yoon H.Y., Sun I.C., Kwon I.C., Kim K. (2020). Tumor-Targeting Glycol Chitosan Nanoparticles for Cancer Heterogeneity. Adv. Mater..

[24] Uchegbu I.F., Sadiq L., Arastoo M., Gray A.I., Wang W., Waigh R.D., Schätzlein A.G. (2001). Quaternary Ammonium Palmitoyl Glycol Chitosan-a New Polysoap for Drug Delivery. Int. J. Pharm..

[25] Odunze U., O’Brien F., Godfrey L., Schätzlein A., Uchegbu I. (2019). Unusual Enthalpy Driven Self Assembly at Room Temperature with Chitosan Amphiphiles. Pharm. Nanotechnol..

[26] Qu X., Khutoryanskiy V.V., Stewart A., Rahman S., Papahadjopoulos-Sternberg B., Dufes C., McCarthy D., Wilson C.G., Lyons R., Carter K.C. (2006). Carbohydrate-Based Micelle Clusters Which Enhance Hydrophobic Drug Bioavailability by up to 1 Order of Magnitute. Biomacromolecules.

[27] Chong W.M., Kadir E.A. (2021). A Brief Review on Hydrophobic Modifications of Glycol Chitosan into Amphiphilic Nanoparticles for Enhanced Drug Delivery. Sains Malays..

[28] Kanwal U., Bukhari N.I., Raza A., Hussain K., Abbas N. (2019). Quaternary Ammonium Palmitoyl Glycol Chitosan-Based Nano-Doxorubicin Delivery System: Potential Applications for Cancer Treatment and Theranostic.

[29] López-Dávila V., Magdeldin T., Welch H., Dwek M.V., Uchegbu I., Loizidou M. (2016). Efficacy of DOPE/DC-Cholesterol Liposomes and GCPQ Micelles as AZD6244 Nanocarriers in a 3D Colorectal Cancer in Vitro Model. Nanomedicine.

[30] Raza A., de la Fuente M., Uchegbu I.F., Schätzlein A. (2010). Modified Glycol Chitosan Nanocarriers Carry Hydrophobic Materials into Tumours. Nanotechnology.

[31] Kanwal U., Bukhari N.I., Rana N.F., Rehman M., Hussain K., Abbas N., Mehmood A., Raza A. (2019). Doxorubicin-Loaded Quaternary Ammonium Palmitoyl Glycol Chitosan Polymeric Nanoformulation: Uptake by Cells and Organs. Int. J. Nanomed..

[32] Cseh K., Geisler H., Stanojkovska K., Westermayr J., Brunmayr P., Wenisch D., Gajic N., Hejl M., Schaier M., Koellensperger G. (2022). Arene Variation of Highly Cytotoxic Tridentate Naphthoquinone-Based Ruthenium(II) Complexes and In-Depth In Vitro Studies. Pharmaceutics.

[33] Göschl S., Schreiber-Brynzak E., Pichler V., Cseh K., Heffeter P., Jungwirth U., Jakupec M.A., Berger W., Keppler B.K. (2017). Comparative Studies of Oxaliplatin-Based Platinum(IV) Complexes in Different in Vitro and in Vivo Tumor Models. Metallomics.

[34] Lalatsa A., Lee V., Malkinson J.P., Zloh M., Schätzlein A.G., Uchegbu I.F. (2012). A Prodrug Nanoparticle Approach for the Oral Delivery of a Hydrophilic Peptide, Leucine5-Enkephalin, to the Brain. Mol. Pharm..

[35] Zia N., Iqbal Z., Raza A., Zia A., Shafique R., Andleeb S., Walker G.C. (2022). Glycol-Chitosan-Based Technetium-99m-Loaded Multifunctional Nanomicelles: Synthesis, Evaluation, and In Vivo Biodistribution. Nanomaterials.

