# Supplementary material for: Quaternary Ammonium Palmitoyl Glycol Chitosan (GCPQ) Loaded with Platinum-Based Anticancer Agents—A Novel Polymer Formulation for Anticancer Therapy
Source: Pharmaceuticals (Basel). 2023 Jul 19;16(7):1027. doi: 10.3390/ph16071027 (PMC10386324; doi:10.3390/ph16071027)
Supplement: Supplementary file 1 [file pharmaceuticals-16-01027-s001.zip › pharmaceuticals-2490417-supplementary.pdf]

Supporting Information

Quaternary ammonium palmitoyl glycol chitosan (GCPQ) loaded with platinum-based anticancer agents – a novel polymer formulation for anticancer therapy

Yvonne Lerchbammer-Kreith [1], Michaela Hejl [1], Nadine S. Sommerfeld [1], Xian Weng-Jiang [2], Uchechukwu Odunze [2], Ryan D. Mellor [2], David G. Workman [2],Michael A. Jakupec[1,3], Andreas G. Schätzlein [2], Ijeoma F. Uchegbu [2,*], Mathea S. Galanski [1,*], Bernhard K. Keppler [1,3]

[1]University of Vienna, Faculty of Chemistry, Institute of Inorganic Chemistry, Waehringer Strasse 42, 1090 Vienna, Austria

[2]University College London, School of Pharmacy, Brunswick Square 29-39, WC1N 1AX London, United Kingdom

[3]Research Cluster "Translational Cancer Therapy Research", University of Vienna, Waehringer Strasse 42, 1090 Vienna, Austria

*Corresponding authors: E-mail addresses: ijeoma.uchegbu@ucl.ac.uk (I.F.U.); mathea.galanski@univie.ac.at (M.S.G.)

Table of Contents

1. ^{1}H NMR spectrum of GCPQ polymer 3
2. Selected NMR spectra of conjugates 4
3. Concentration-effect curves........ 8
4. Solubility data 12
5. References 13

1. ¹H NMR spectrum of GCPQ polymer

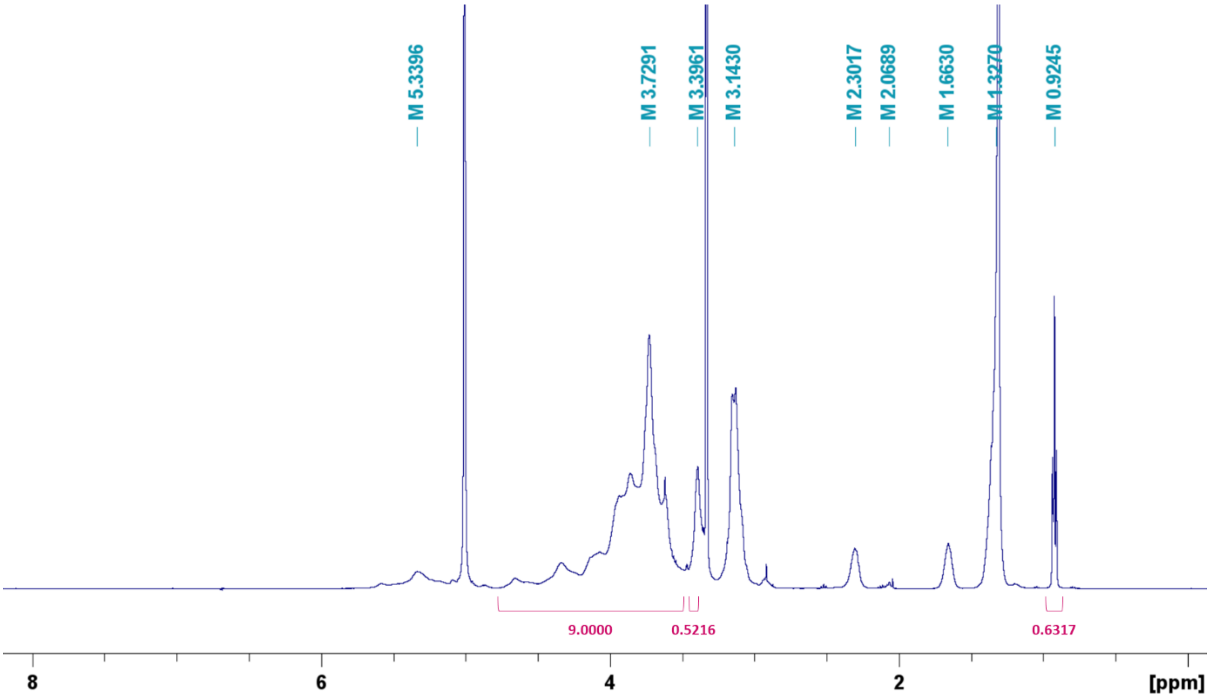

Figure S1. ¹H NMR spectrum of polymer **GCP21Q12** in MeOD + 5μL DCl. The levels of palmitoylation and quaternisation were estimated by comparison of the integrals of palmitoyl methyl protons (0.92 ppm, 3H), quaternary ammonium protons (3.40 ppm, 9H, due to the partly overlapping MeOD signal, only half of the quaternary ammonium signal was integrated and accounted as 4.5 protons, provided a symmetrical resonance) and sugar backbone protons (3.54–4.75 ppm, 9H), respectively [1] .

2. Selected NMR spectra of conjugates

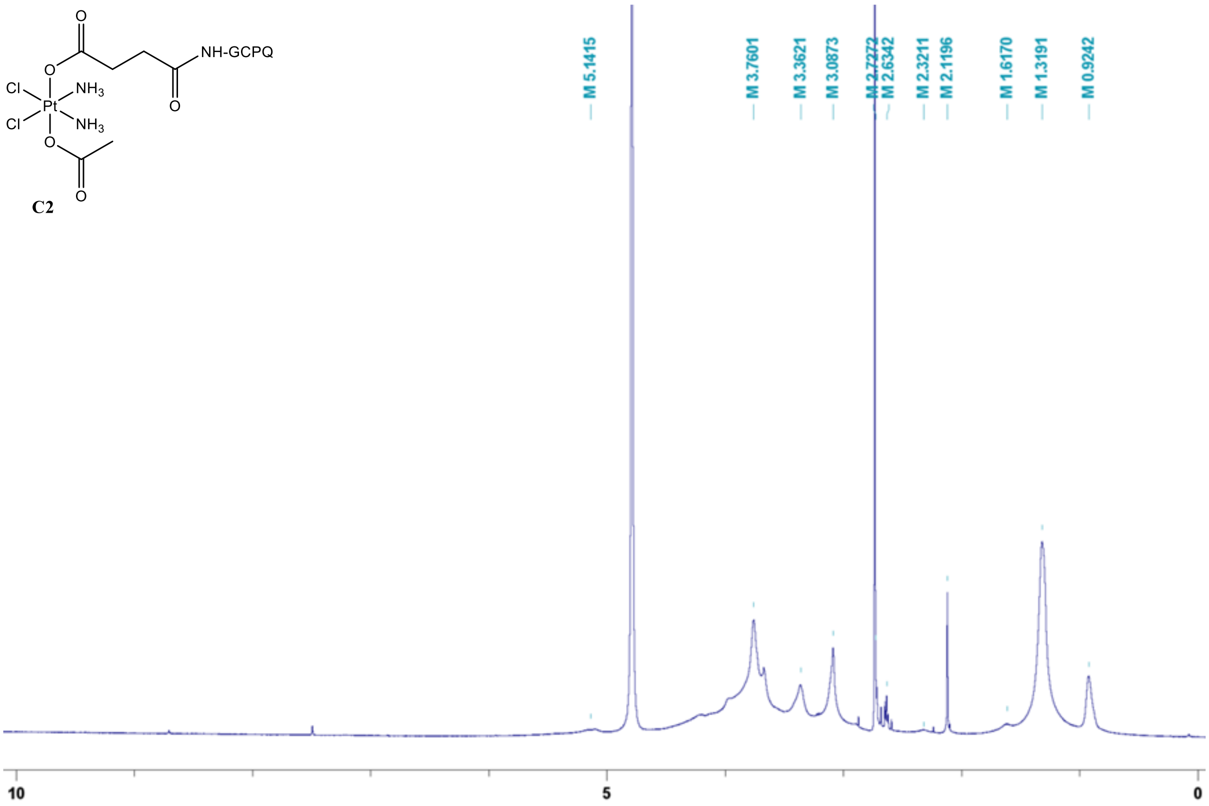

Figure S2. ^{1}H NMR spectrum of conjugate **C2** in D_2O

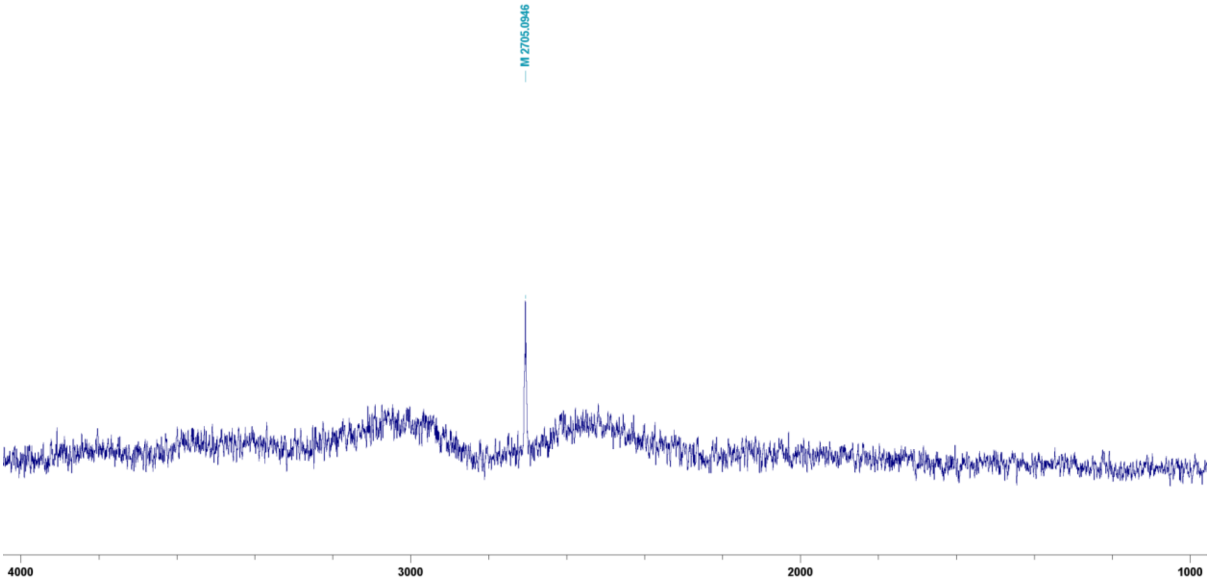

Figure S3. ^{195}Pt NMR spectrum of conjugate **C2** in D_2O.

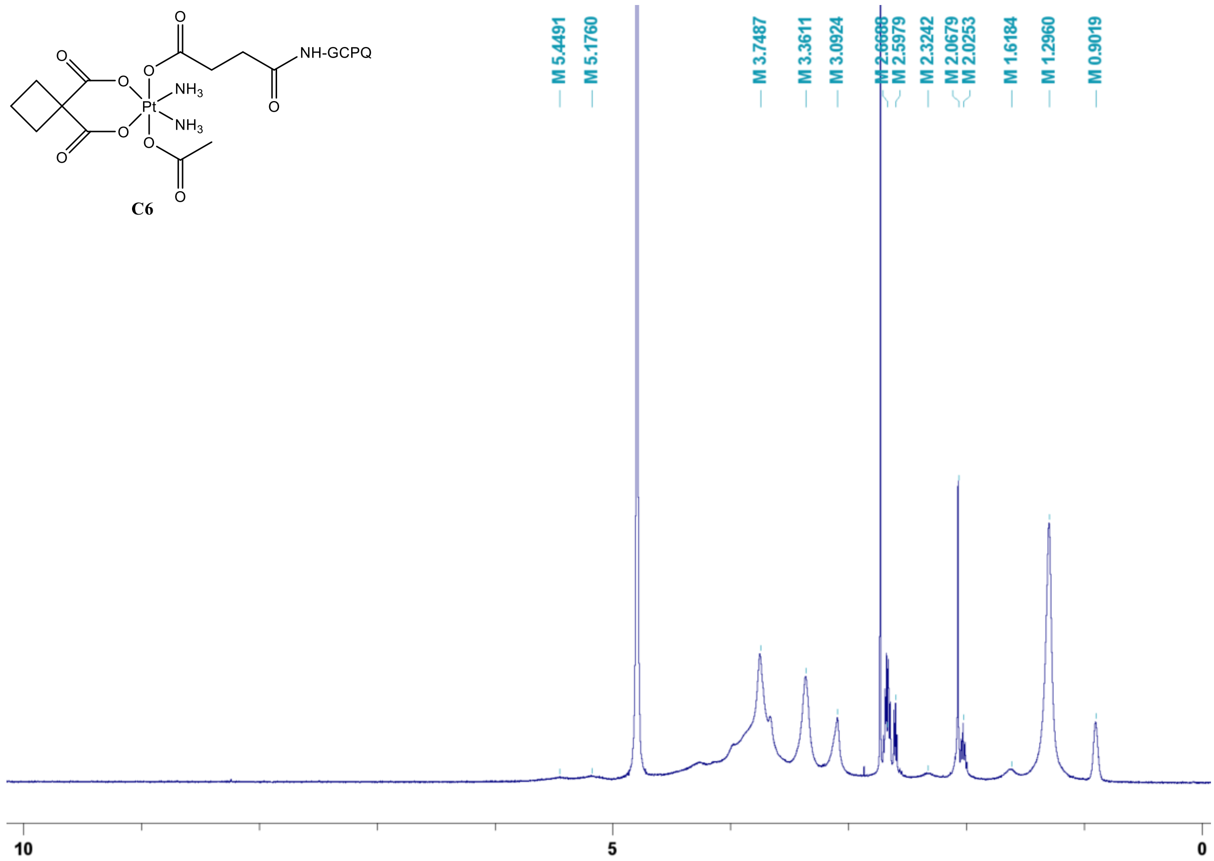

Figure S4. ^{1}H NMR spectrum of conjugate **C6** in D_2O.

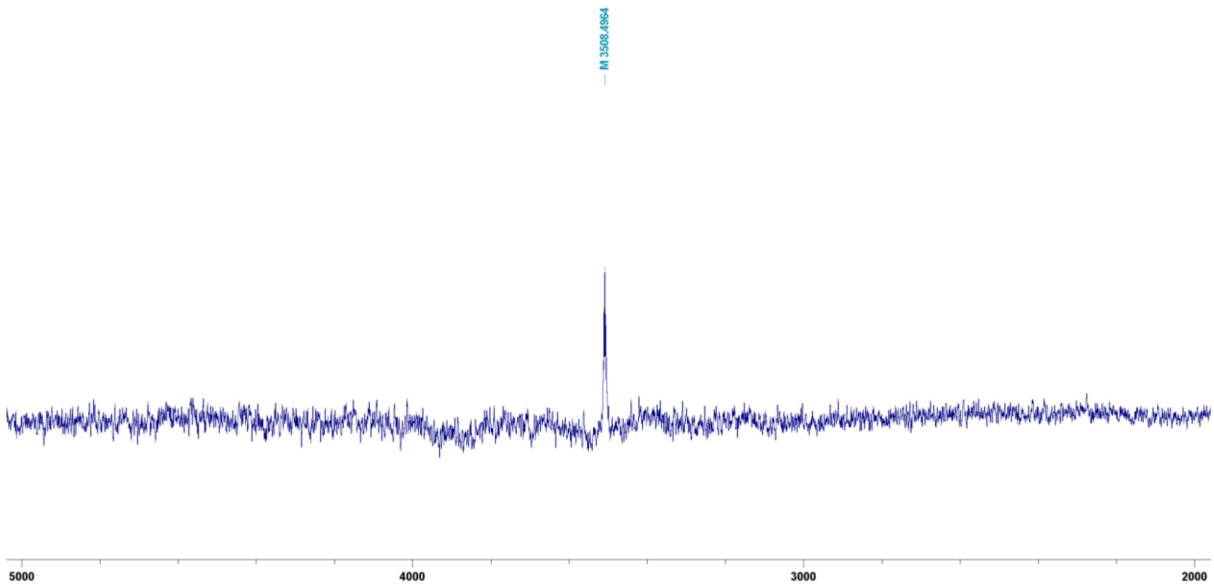

Figure S5. ^{195}Pt NMR spectrum of conjugate **C6** in D_2O.

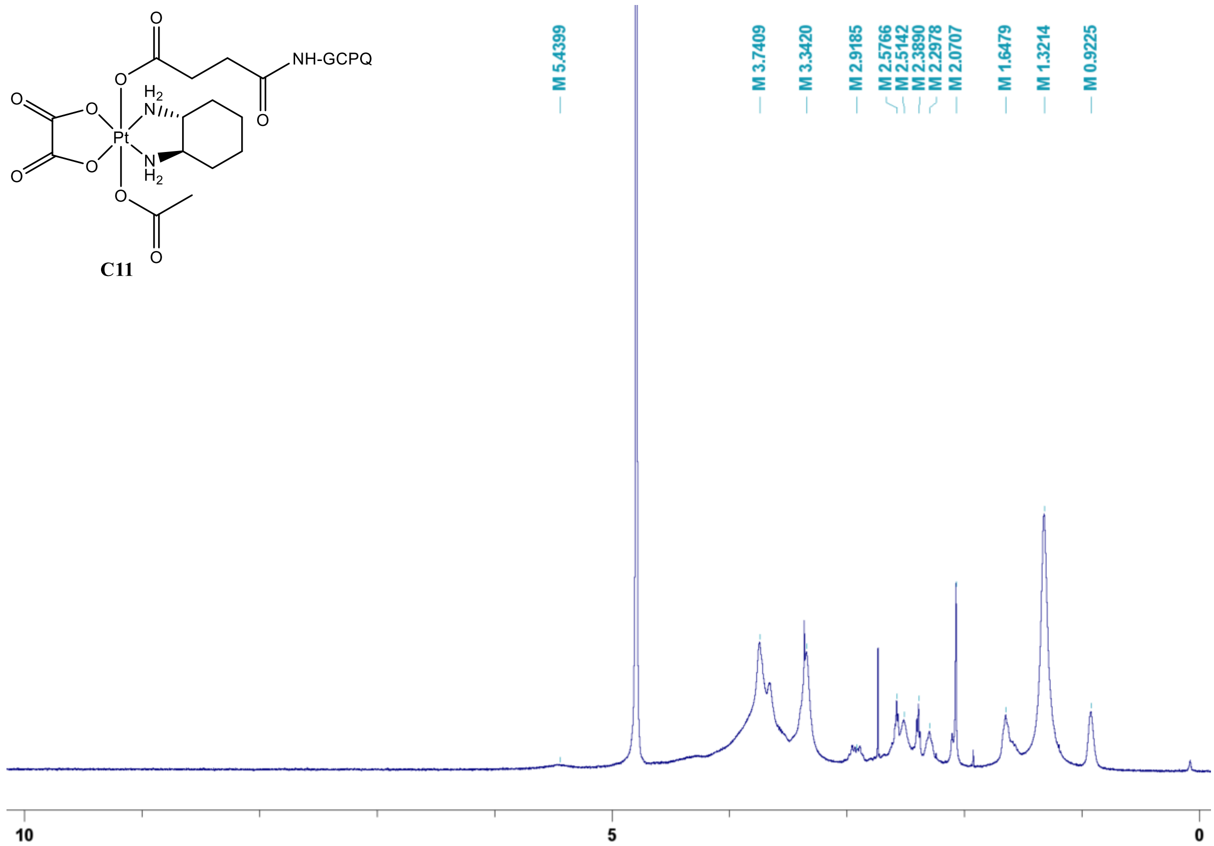

Figure S6. ^{1}H NMR spectrum of conjugate **C11** in D_2O.

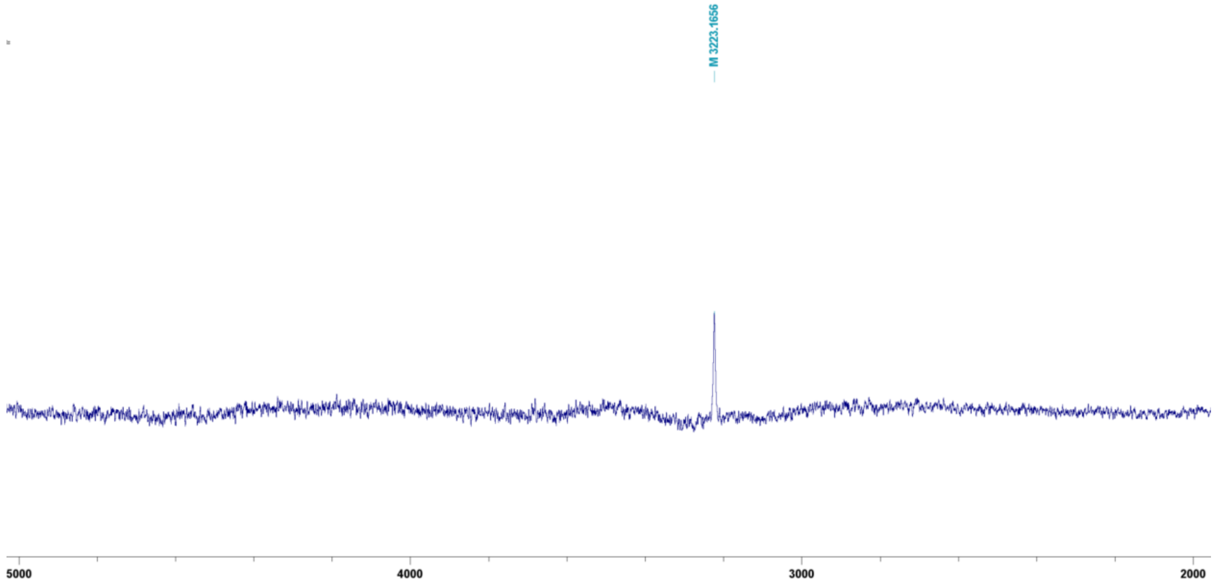

Figure S7. ^{195}Pt NMR spectrum of conjugate **C11** in D_2O.

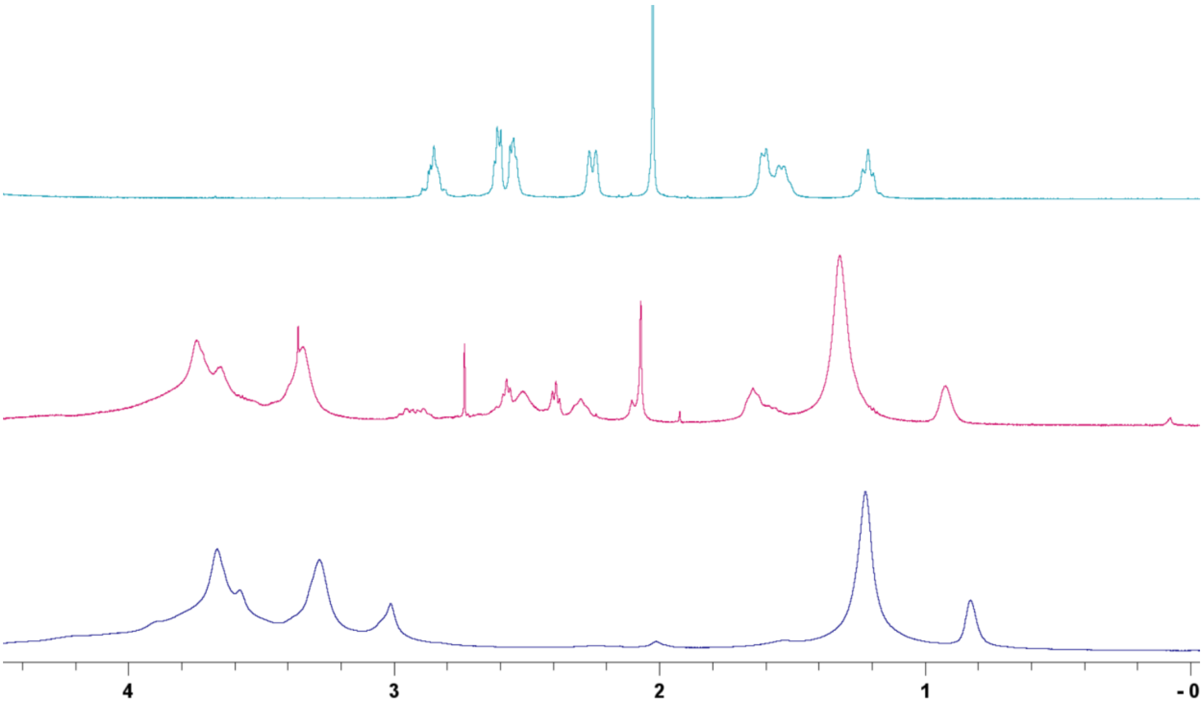

Figure S8. ¹H NMR spectra measured in D_2O of platinum(IV) complex **3** (above), conjugate **C11** (middle) and **GCP22Q33** polymer (below).

3. Concentration-effect curves

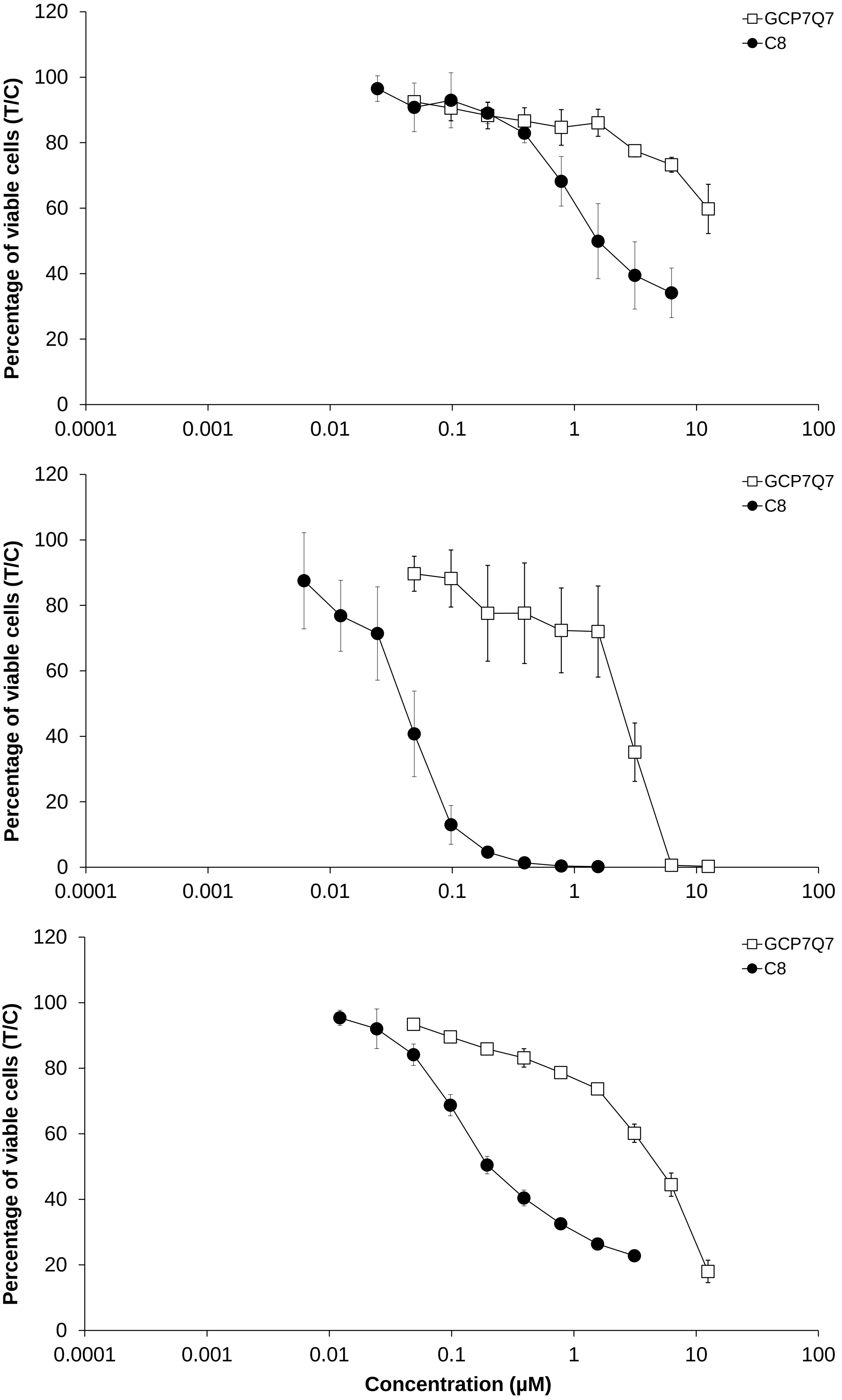

Figure S9. Concentration-effect curves of **GCP7Q7** and **C8** in A549 (top), CH1/PA-1 (middle) and SW480 (bottom) cells, obtained by MTT assays with 96 h exposure time. Values are means ± standard deviations from at least three independent experiments.

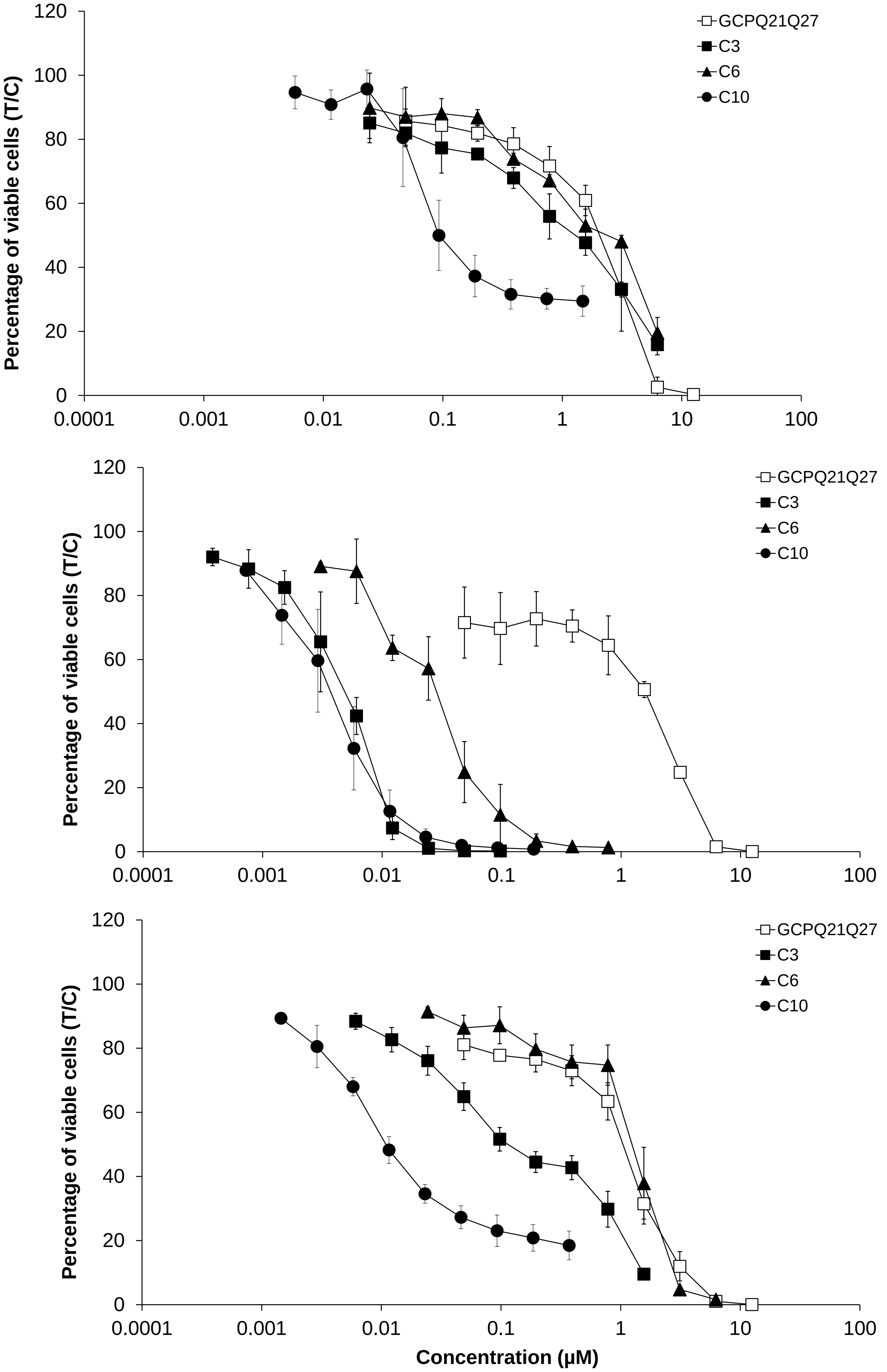

Figure S10. Concentration-effect curves of **GCPQ21Q27**, **C3**, **C6** and **C10** in A549 (top), CH1/PA-1 (middle) and SW480 (bottom) cells, obtained by MTT assays with 96 h exposure time. Values are means ± standard deviations from at least three independent experiments.

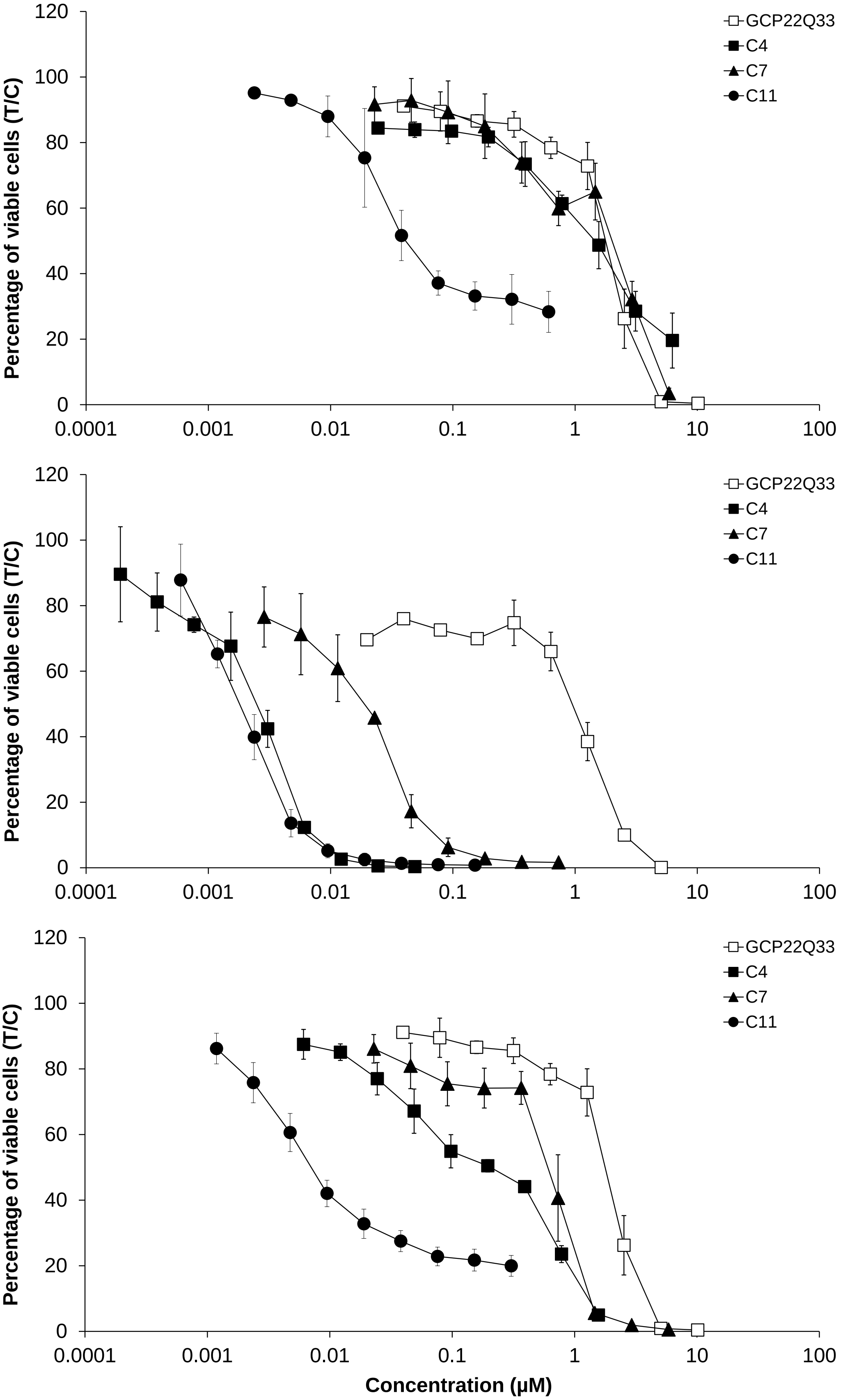

Figure S11. Concentration-effect curves of **GCP22Q33**, **C4**, **C7** and **C11** in A549 (top), CH1/PA-1 (middle) and SW480 (bottom) cells, obtained by MTT assays with 96 h exposure time. Values are means ± standard deviations from at least three independent experiments.

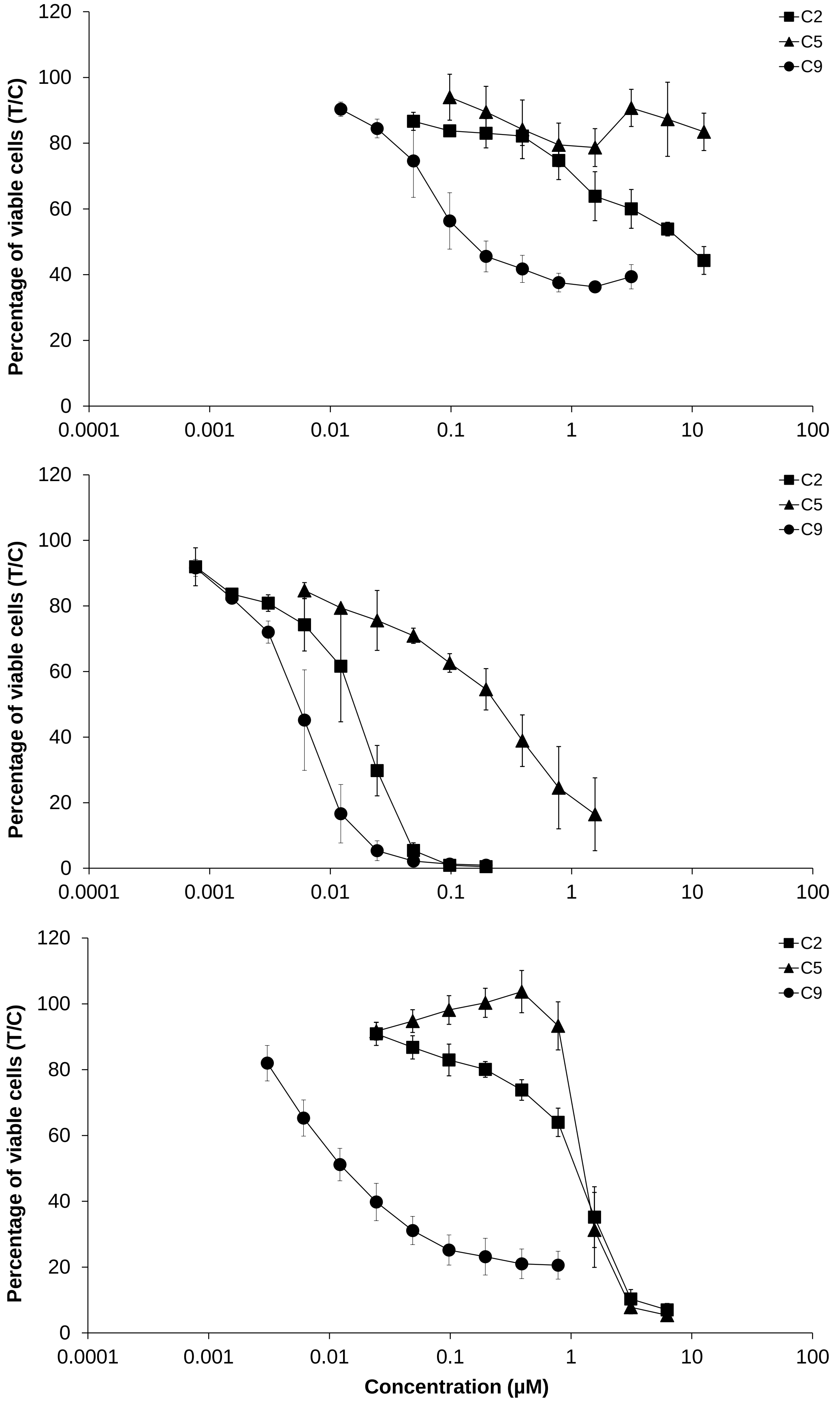

Figure S12. Concentration-effect curves of **C2**, **C5** and **C9** in A549 (top), CH1/PA-1 (middle) and SW480 (bottom) cells, obtained by MTT assays with 96 h exposure time. Values are means ± standard deviations from at least three independent experiments.

4. Solubility data

Table S1. Overview of the water solubility of conjugates **C1**-**C11** measured by visual judgement in Milli-Q water at room temperature.

Sample	Pt(IV)	GCPQ	Water solubility [mg/mL]
**C1**	**1**	**GCP7Q7**	~1
**C2**	**1**	**GCP21Q12**	~3
**C3**	**1**	**GCP21Q27**	~4
**C4**	**1**	**GCP22Q33**	~5.5
**C5**	**2**	**GCP21Q12**	~3
**C6**	**2**	**GCP21Q27**	~5
**C7**	**2**	**GCP22Q33**	~6
**C8**	**3**	**GCP7Q7**	~2
**C9**	**3**	**GCP21Q12**	~4
**C10**	**3**	**GCP21Q27**	~5
**C11**	**3**	**GCP22Q33**	~6.5

5. References

1. Uchegbu, I.F.; Sadiq, L.; Arastoo, M.; Gray, A.I.; Wang, W.; Waigh, R.D.; Schätzlein, A.G. Quaternary Ammonium Palmitoyl Glycol Chitosan-a New Polysoap for Drug Delivery. *Int J Pharm* **2001**, *224*, 185–199, doi:10.1016/s0378-5173(01)00763-3.
